# Edoxaban for stroke prevention in Chinese patients with atrial fibrillation: 1-year follow-up of the ETNA-AF-China study

**DOI:** 10.3389/fphar.2026.1739246

**Published:** 2026-01-22

**Authors:** Xueyuan Guo, Juan Du, Yuehui Yin, Heng Qi, Zhisheng Jia, Xiaojun Ji, Yuqing Zhang, Xue Liang, Bing Deng, Jieyun Liu, Juan Ma, Cangsang Song, Huifang Feng, Suxin Luo, Jingfeng Wang, Yongqi Xiao, Lun Li, Junyou Cui, Zheng Huang, Xiang Cheng, Yuan Yi, Mengqi Liu, Cathy Chen, Martin Unverdorben, Changsheng Ma

**Affiliations:** 1 Beijing Anzhen Hospital, Capital Medical University, Beijing, China; 2 Daiichi Sankyo (China) Holdings Co., Ltd, Shanghai, China; 3 The Second Affiliated Hospital of Chongqing Medical University, Chongqing, China; 4 The First Hospital of Changsha, Changsha, China; 5 The Fifth People’s Hospital of Jinan, Jinan, China; 6 Wenzhou Central Hospital, Wenzhou, China; 7 Nanjing Jiangning Hospital, Nanjing, China; 8 The Fifth Affiliated Hospital of Zhengzhou University, Zhengzhou, China; 9 Longhua Hospital Shanghai University of Traditional Chinese Medicine, Shanghai, China; 10 Kaifeng Central Hospital, Kaifeng, China; 11 The First Hospital of Kunming, Kunming, China; 12 Taiyuan Central Hospital of Shanxi Medical University, Taiyuan, China; 13 The First Affiliated Hospital of Chongqing Medical University, Chongqing, China; 14 Sun Yat-sen Memorial hospital, Sun Yat-sen University, Guangzhou, China; 15 The Third People’s Hospital of Nanning, Nanning, China; 16 Wuhan Puai Hospital, Wuhan, China; 17 Jiangyin People’s Hospital, Wuxi, China; 18 The First Affiliated Hospital of Guangzhou Medical University, Guangzhou, China; 19 Union Hospital Affiliated with Tongji Medical College of Huazhong University of Science and Technology, Wuhan, China; 20 Daiichi Sankyo Inc., Basking Ridge, NJ, United States

**Keywords:** atrial fibrillation, Chinese, edoxaban, non–vitamin K antagonist oral anticoagulants, real-world

## Abstract

**Introduction:**

Non–vitamin K antagonist oral anticoagulants (NOACs) are the first-line therapy to prevent ischaemic stroke in patients with atrial fibrillation (AF). However, studies on the effectiveness and safety of edoxaban for Chinese patients with AF, are limited.

**Methods:**

We report the 1-year interim follow-up data on edoxaban use in Chinese patients with AF from the ETNA-AF-China (NCT04747496), a multicentre, prospective, observational study conducted in 89 centres across the Chinese Mainland, enrolling 5,001 patients with a total of 2-year follow-up. No adjustment for multiple testing was made; therefore, all *P* values must be interpreted in an exploratory or descriptive manner.

**Results:**

Overall, 4,877 patients (60 mg edoxaban: 54.3%; 30 mg edoxaban: 45.7%) completed 1-year follow-up (mean age ± standard deviation: 70.3 ± 9.5 years; mean CHA_2_DS_2_-VASc score: 2.9 ± 1.4: mean HAS-BLED score: 1.8 ± 1.0). All-cause death occurred in 100 patients (annualised event rate: 2.30%/y), of whom 27 (0.62%/y) died from cardiovascular (CV) events. Annualised rates for major bleeding (45 patients [1.04%/y]), intracranial hemorrhage (ICH, nine patients [0.21%/y]), and major gastrointestinal bleeding (19 patients [0.44%/y]) were low. Patients receiving edoxaban 30 mg had numerically higher rates of all-cause death, CV death, and major bleeding than edoxaban 60 mg (*P* < 0.05), potentially because of diverse baseline characteristics. Lower BMI, permanent AF type, history of major bleeding, and frailty identified as risk factors of all-cause death by multivariable Cox analysis. After 1 year, 73.1% patients continued edoxaban use without suspension, discontinuation of edoxaban, or switching to other doses of edoxaban/other NOACs.

**Conclusion:**

In a large Chinese AF population, edoxaban showed low incidences of stroke and bleeding, notably major bleeding, ICH, major gastrointestinal bleeding, and CV mortalities, with the majority of patients still on edoxaban at the end of 1-year follow-up.

## 1 Introduction

Atrial fibrillation (AF) is the most common type of cardiac arrhythmia, affecting 2%–4% of adults globally (Benjamin et al., 2019; Shi et al., 2022), with an estimated prevalence of 1.6% among Chinese adults (Shi et al., 2022). Globally, the prevalence of AF has increased over the past 2 decades and is expected to continue growing for the next three decades, posing one of the largest public health challenges (Lippi et al., 2021). Recent studies have shown a rapid increase in the prevalence and the economic burden of AF in China, largely driven by an aging population, socio-economic growth, and a rising prevalence of its associated risk factors, including overweight or obesity (Sun et al., 2023). In 2021, the global prevalence of stroke was estimated at 93.8 million (Feigin et al., 2025), with ∼17.8 million adults affected in China alone (Tu et al., 2023). Despite this, anticoagulation used among adults remains suboptimal. In Chinese community settings, AF management has seen limited progress, with oral anticoagulants being prescribed to only a small proportion (∼5%) of patients at high risk of stroke (Jiang et al., 2024). These gaps underscore the urgent need for regionally tailored analysis and a comprehensive strategy that integrates prevention and treatment of stroke (Tu et al., 2023).

Currently, non-vitamin K antagonist (VKA) oral anticoagulants (NOACs) are recommended as the first-choice treatment for preventing stroke and systemic embolism events (SEEs) in patients with AF (Hindricks et al., 2021; Chinese Society of Cardiology, Chinese Medical Association and Heart Rhythm Committee of Chinese Society of Biomedical Engineering, 2023; Schnabel et al., 2023; European Medicines Agency, 2024). Edoxaban, with the rapid onset mechanism of factor Xa direct inhibition, is administered orally at 60 mg once daily (OD) and with a reduced dose of 30 mg OD for patients with renal impairment (creatine clearance [CrCl] 15–50 mL/min, low body weight [≤60 kg] or using specific P-glycoprotein [P-gp] inhibitors) by label recommendation (European Medicines Agency, 2024). Its antithrombotic efficacy was demonstrated in phase 3 of the ENGAGE AF-TIMI 48 trial reported, which showed reduced stroke or SEE events and lower rates of bleeding and cardiovascular (CV) death than warfarin in the non-valvular atrial fibrillation (NVAF) population (Giugliano et al., 2013). Based on this evidence, edoxaban was approved by the US Food and Drug Administration (FDA) (SAVAYSA, 2015), the European Medicines Agency (EMA), the Ministry of Health, Labour and Welfare (MHLW) in Japan, and the Korean Ministry of Food and Drug Safety (MFDS) in South Korea (Choi et al., 2021), for the prevention of ischemic stroke or SEE in AF. In China, edoxaban was approved in 2018 and added to the National Reimbursement Drug List in 2020 (Zhang et al., 2022). However, there is still limited real-world evidence on the clinical management of edoxaban use in the Chinese population with AF, and optimizing CV risk factors as part of an integrated approach to AF management (Chinese Society of Cardiology, Chinese Medical Association and Heart Rhythm Committee of Chinese Society of Biomedical Engineering, 2023; Schnabel et al., 2023; Joglar et al., 2024).

ETNA-AF-China is one of the Global Edoxaban Treatment in rouTine clinical prActice for patients with nonvalvular Atrial Fibrillation (ETNA-AF) programme, a large-scale noninterventional study that stands as the most extensive noninterventional study focusing on a single direct oral anticoagulant in clinical practice of AF with overall 2-year follow-up (De Caterina et al., 2019b; De Caterina et al., 2019a; De Caterina et al., 2021). The current analysis reports the 1-year interim cut-off data of the ongoing ETNA-AF-China study on the effectiveness and safety of edoxaban in Chinese patients with AF.

## 2 Methods

### 2.1 Study design

ETNA-AF-China (Clinicaltrials.gov: NCT04747496) is a multicentre, prospective, observational study designed to evaluate the effectiveness and safety of edoxaban for stroke prevention in 89 sites across the Chinese Mainland, enrolling 5,001 patients with a total of 2-year follow-up. ETNA-AF-China included three predefined data cut-off points for analysis: baseline, 1-year outcomes, and final 2-year outcomes, based on scheduled follow-up data collection. Here, we present the first 1-year follow-up outcomes based on the interim data cut-off on 1 February 2024; data are subject to change as data collection was ongoing at the time of interim data cut-off. Final data cleaning for 2-year follow-up is ongoing. The detailed study design and rationale of the Global ETNA-AF programme, and baseline characteristics of ETNA-AF-China were previously published (De Caterina et al., 2019b; De Caterina et al., 2019a; Guo et al., 2024).

### 2.2 Patient population

Patients with a history of NVAF who were prescribed edoxaban at enrolment or baseline (≤1 week after baseline) and did not simultaneously participate in an interventional trial were eligible upon providing written consent. No exclusion criteria were present to avoid any possible selection bias.

### 2.3 Objectives, definitions, and outcome measures

The primary objective of the study was to evaluate the real-world safety of edoxaban by evaluating bleeding events (including major, major or clinically relevant non-major [CRNM] bleeding according to the International Society on Thrombosis and Haemostasis’s [ISTH] definition) (Schulman and Kearon, 2005) intracranial haemorrhage [ICH], all-cause and CV mortality in patients with AF. Stroke (ischaemic, haemorrhagic, and unknown), SEEs, transient ischaemic attack (TIA), and myocardial infarction (MI) were evaluated as secondary outcomes.

The CHA_2_DS_2_-VASc and HAS-BLED scores were both centrally calculated based on the investigator-reported baseline clinical characteristics of patients. The DOAC score was calculated based on the development and validation by R. Aggarwal (Aggarwal et al., 2023). The ‘recommended dose’ for edoxaban treatment is 60 mg OD as labelled and 30 mg OD for those who met at least one of the following criteria: (1) moderate-severe renal impairment (CrCl ≤50 mL/min), (2) body weight ≤60 kg and (3), concomitant use of label-listed P-gp inhibitors. “Non-recommended dose” is referred to as not in line with label recommendation, that is, 60 mg was considered non-recommended if the patient fulfilled at least one dose reduction criterion, or 30 mg was non-recommended if the patient did not meet any dose reduction criterion.

### 2.4 Statistical analysis

Statistical analyses were performed on the full analysis set (FAS). FAS was defined as all patients with any documentation (such as post-baseline ADRs, AF-related clinical events, or hospitalisations) from at least one follow-up data collection or at final assessment (any information except “lost to follow-up”) from BAS.

Baseline analysis set (BAS) was defined as: (1) the patient with a confirmed history of NVAF by an electrical test (e.g., electrocardiogram, Holter monitoring, pacemaker, or implantable cardioverter-defibrillator). (2) The investigator confirmed that the patient was receiving treatment with edoxaban after providing informed consent. In addition, those patients who were judged by the investigator as not receiving edoxaban treatment after providing informed consent were additionally checked whether edoxaban initiation date was at least within 7 days after baseline visit date. (3) The patient not concurrently participating in any other interventional studies was included (Supplementary Figure S1).

The baseline characteristics were summarised descriptively as frequencies (“n” and percentage) and mean value ± standard deviation (SD) rounded to an integer. Patient characteristics between the 60 mg and 30 mg dose groups were compared using the chi-square test for the categorical data and the Mann–Whitney *U* test for the continuous data. The normality of continuous data was checked using the Shapiro test. Adherence to label-recommendation (recommended dose/non-recommended dose) was calculated by dose prescription at baseline.

Based on the reported events of the investigators, annualised event rates of the safety and effectiveness outcomes were presented. No preplanned central adjudication of this real-world study was carried out; all events were reported by qualified investigators. Annualised event rates were estimated by censoring those lost to follow-up or premature termination. Annual rates were based on time exposure of whole population regardless of the discontinuation of edoxaban or edoxaban dose. The annualised event rates were calculated as 100*[number of patients with at least one event within 2 years after baseline] divided by [the sum of all individual patient observation time]. Individual patient observation time was defined as time in years from baseline data collection point (i.e., reference start date) until first event date (within 2 years after baseline), or if patient completed/discontinued from the study without experiencing an event (within 2 years after baseline), until the date of premature study discontinuation, or death, or last available follow-up date, or censoring after 24 months from baseline data collection point whichever occurred first.

Kaplan–Meier curves were used to present the cumulative event rates of all stroke/SEE/TIA, major bleeding, and all-cause mortality. Two or more time-to-event data points of the curves were compared using the log-rank test. Univariable Cox regression analysis identified the hazard ratio (HR) and 95% confidence interval (CI) for recommended or non-recommended doses and 30 mg versus 60 mg related outcomes.

Multivariable Cox model was conducted using a stepwise regression method by the Akaike Information Criterion for all covariables while fixing edoxaban dosing (60 mg and 30 mg) and recommended/non-recommended doses. The prespecified variables included edoxaban dose, age category, gender, body mass index (BMI) category, creatinine clearance category, economic region, CHA_2_DS_2_-VASc score, modified HAS-BLED score, AF type, frailty, chronic obstructive pulmonary disease, prior TIA, hypertension, heart failure, diabetes, dyslipidaemia, valvular heart disease, prior stroke at baseline, and prior major bleeding.

Statistical analyses were performed using R Statistical Software (version 4.3.2, Boston, Massachusetts, United States). No adjustment for multiple testing was made; therefore, all P values must be interpreted in a purely exploratory or descriptive way.

### 2.5 Ethical standards

The study was approved by the institutional review boards and independent Ethics Committees from Beijing Anzhen Hospital (No. 2019-058D) and all participating centres in compliance with the Declaration of Helsinki and Guidelines for Good Pharmacoepidemiology Practice (GPP) (Public Policy Committee, International Society of Pharmacoepidemiology, 2016). All participants provided written informed consent.

## 3 Results

### 3.1 Baseline characteristics

Of the overall 5,001 patients enrolled in the study, 124 patients were excluded because of either receiving unspecified doses, starting edoxaban more than 1 week after baseline, participating simultaneously in other interventional studies, or being lost to follow-up without any post-baseline documentation (Supplementary Figure S1). The baseline demographics and clinical characteristics of the FAS, consisting of 4,877 patients, with 2,650 patients (54.3%) initially received edoxaban 60 mg and 2,227 (45.7%) received 30 mg, are presented in Table 1. The overall mean (SD) patient age was 70.3 ± 9.5 years, and 2,780 patients (57.0%) were male. The majority of the patient population belonged to the Eastern economic region (49.9%), followed by the Middle (33.5%), Northeastern (8.6%), and Western economic regions (8.1%) in China. The mean (SD) body weight was 67.9 ± 12.5 kg, and the mean (SD) body mass index (BMI) was 25.0 ± 3.7 kg/m^2^. The calculated mean (SD) CrCl by the Cockcroft–Gault formula was 71.0 ± 27.2 mL/min, and the mean (SD) estimated glomerular filtration rate (eGFR) by CrCl normalized to 1.73 m^2^ of body surface area using the Du Bois and Du Bois formula (Michels et al., 2010) was 58.7 ± 19.8 mL/min/1.73 m^2^. The calculated mean (SD) CHA_2_DS_2_-VASc and modified HAS-BLED scores were 2.9 ± 1.4 and 1.8 ± 1.0, respectively.

**TABLE 1:** Baseline demographics and clinical characteristics for the overall population.

Baseline characteristics	Total [*n* = 4,877]^a^ (100%)	60 mg [*n* = 2,650] (54.3%)	30 mg [*n* = 2,227] (45.7%)	*P* value
Male, *n* (%)	2,780 (57.0)	1812 (68.4)	968 (43.5)	<0.001
Age (years), mean (SD)	70.3 (9.5)	67.6 (9.1)	73.4 (9.0)	<0.001
By age sub-groups, *n* (%)	​	​	​	<0.001
<65 years	1,138 (23.3)	820 (30.9)	318 (14.3)	-
≥65–<75 years	2039 (41.8)	1,232 (46.5)	807 (36.2)	-
≥75–<85 years	1,468 (30.1)	556 (21.0)	912 (41.0)	-
≥85 years	232 (4.8)	42 (1.6)	190 (8.5)	-
Body weight^b^ (kg), mean (SD)	67.9 (12.5)	73.3 (11.0)	61.5 (11.1)	<0.001
BMI^c^ (kg/m^2^), mean (SD)	25.0 (3.7)	26.2 (3.4)	23.5 (3.4)	<0.001
By BMI subgroups, n (%)	​	​	​	<0.001
<18.5 kg/m^2^	130 (2.7)	6 (0.2)	124 (5.6)	​
≥18.5–< 25 kg/m^2^	2,367 (48.5)	991 (37.4)	1,376 (61.8)	​
≥25 kg/m^2^	2,273 (46.6)	1,623 (61.2)	650 (29.2)	​
(calc) creatinine clearance^d^ (mL/min), mean (SD)	71.0 (27.2)	81.5 (26.2)	59.2 (23.2)	<0.001
By CrCl subgroups, *n* (%)	​	​	​	<0.001
≥80 mL/min	1,262 (25.9)	962 (36.3)	300 (13.5)	-
≥50–<80 mL/min	1865 (38.2)	1,002 (37.8)	863 (38.8)	-
≥30–<50 mL/min	709 (14.5)	123 (4.6)	586 (26.3)	-
<30 mL/min	131 (2.7)	10 (0.4)	121 (5.4)	-
(calc) eGFR^e^ (mL/min/1.73 m^2^), mean (SD)	58.7 (19.8)	64.7 (18.6)	51.9 (18.8)	<0.001
(calc) CHA_2_DS_2_-VASc score^f^, mean (SD)	2.9 (1.4)	2.6 (1.3)	3.3 (1.4)	<0.001
(calc) CHA_2_DS_2_-VA score^g^, mean (SD)	2.9 (1.4)	2.6 (1.4)	3.1 (1.5)	<0.001
(mod) HAS-BLED score^h^, mean (SD)	1.8 (1.0)	1.5 (1.0)	2.0 (0.9)	<0.001
DOAC score^i^, mean (SD)	4.3 (2.3)	3.6 (2.1)	5.1 (2.3)	<0.001
Type of AF^j^, *n* (%)	​	​	​	0.02
Paroxysmal	1780 (36.5)	918 (34.6)	862 (38.7)	-
Persistent	1,584 (32.5)	862 (32.5)	722 (32.4)	-
Long-standing persistent	828 (17.0)	484 (18.3)	344 (15.4)	-
Permanent	669 (13.7)	377 (14.2)	292 (13.1)	-
LVEF categorised by 40%^k^	​	​	​	0.09
<40%	163 (6.4)	78 (5.8)	85 (7.1)	-
≥40%	2,379 (93.6)	1,274 (94.2)	1,105 (92.9)	-
Perceived frailty^l^, *n* (%)	309 (6.3)	89 (3.4)	220 (9.9)	<0.001
COPD^m^, *n* (%)	234 (4.8)	109 (4.1)	125 (5.6)	0.02
Hypertension^n^, *n* (%)	3,581 (73.4)	2010 (75.8)	1,571 (70.5)	<0.001
Heart failure (derived)^o^, *n* (%)	716 (14.7)	326 (12.3)	390 (17.5)	<0.001
Diabetes mellitus^p^, *n* (%)	1,284 (26.3)	737 (27.8)	547 (24.6)	0.01
Dyslipidaemia^o^ ^,^ ^q^, *n* (%)	1,225 (25.1)	723 (27.3)	502 (22.5)	<0.001
Valvular disease^r^, *n* (%)	265 (5.4)	118 (4.5)	147 (6.6)	0.001
History of ischaemic stroke, *n* (%)	351 (7.2)	169 (6.4)	182 (8.2)	0.02
History of TIA, *n* (%)	108 (2.2)	57 (2.2)	51 (2.3)	0.82
History of major or CRNM bleeding, *n* (%)	62 (1.3)	32 (1.2)	30 (1.3)	0.76
History of major bleeding, *n* (%)	54 (1.1)	30 (1.1)	24 (1.1)	0.97
Overall adherence to label^s^, *n* (%)	​	​	​	<0.001
Recommended edoxaban dose	3,269 (78.0)	1790 (83.8)	1,479 (71.9)	-
Non-recommended edoxaban dose	923 (22.0)	345 (16.2)	578 (28.1)	-
Economic region, *n* (%)	​	​	​	<0.001
East	2,432 (49.9)	1,447 (54.6)	985 (44.2)	-
Northeast	417 (8.6)	254 (9.6)	163 (7.3)	-
Middle	1,632 (33.5)	768 (29.0)	864 (38.8)	-
West	396 (8.1)	181 (6.8)	215 (9.7)	-

^a^ Based on full analysis set (FAS); all percentages are based on the total number of patients with all observations (including missing and unknown data).

^b^ Body weight was missing for 55 patients.

^c^ BMI, was missing for 107 patients.

^d^ The CrCl calculation was based on the Cockcroft-Gault formula and was missing for 910 patients.

^e^ eGFR, was calculated by the Du Bois and Du Boi method; it was missing for 973 patients.

^f^ Derived variables of the CHA_2_DS_2_-VASc, score, CHA_2_DS_2_-VA, score and HAS-BLED, score. 138 patients had a missing CHA_2_DS_2_-VASc, score.

^g^ 165 patients had a missing CHA_2_DS_2_-VA, score.

^h^ The HAS-BLED, score was calculated without ‘labile INR’; the renal impairment item was derived instead of investigator reported; alcohol abuse was defined as >1 drink/d; missing for 565 patients.

^i^ DOAC, score was missing for 539 patients.

^j^ AF, type was missing for 16 patients.

^k^ LVEF, data were missing for 2,335 patients (1,298 and 1,037 patients in the edoxaban 60 mg and 30 mg groups, respectively).

^l^ Perceived frailty was missing for 867 patients.

^m^ COPD, was missing for 87 patients.

^n^ Hypertension was unknown in 22 patients.

^o^ Patients were considered as heart failure with documented congestive HF, documented ischaemic cardiomyopathy, LVEF <40%, or frequent dyspnoea (≥1/d) without COPD, and at least one of the following: documented severe valvular heart disease, documented CAD, post-myocardial infarction, valve replacement or documented hypertension treated with at least three drugs.

^p^ Diabetes mellitus was unknown for 35 patients.

^q^ Dyslipidaemia was unknown for 123 patients.

^r^ Valvular disease was unknown for 114 patients.

^s^ Recommended doses were calculated based on the percentage of judgeable patients (*n* = 4,192); 685 patients were non-judgable dosing. *P* values were calculated by comparing 30 mg and 60 mg dose levels.

AF, atrial fibrillation; BMI, body mass index; COPD, chronic obstructive pulmonary disease; CrCl, creatinine clearance; CRNM, clinically relevant non-major; eGFR, estimated glomerular filtration rate; LVEF, left ventricular ejection fraction; TIA, transient ischaemic attack; SD, standard deviation.

Most of the patients presented with paroxysmal (1780; 36.5%) and persistent AF (1,548; 32.5%), with only 17% and 13.7% of them having long-standing persistent and permanent AF, respectively. The majority of the patients (2,379; 93.6%) had a left ventricular ejection fraction of ≥40%. The extent of perceived frailty was minimal (309; 6.3%).

Patients receiving 30 mg edoxaban OD at baseline were older (mean 73.4 years vs. 67.6 years) had higher CHA_2_DS_2_-VASc score (3.3 vs. 2.6), CHA_2_DS_2_-VA score (3.1 vs. 2.6), modified HAS-BLED score (2.0 vs. 1.5), DOAC score (5.1 vs. 3.6), and more percentage of impaired renal function (CrCl <80 mL/min: 70.5% vs. 42.8%) than those receiving 60 mg (all *P* < 0.001). When compared with the 60 mg group, the 30 mg group had a higher proportion of patients with perceived frailty (9.9% vs. 3.4%; *P* < 0.001), higher proportion of HF (17.5% vs. 12.3%; *P* < 0.001), and valvular disease (6.6% vs. 4.5%, *P* = 0.001), whereas a smaller percentage of hypertension (70.5% vs. 75.8%, *P* < 0.001) and dyslipidaemia (22.5% vs. 27.3%, *P* < 0.001) (Supplementary Table S1; Supplementary Figure S2).

The overall adherence of edoxaban to the label recommendation was high (3,269/4,192 [with evaluable data]; 78.0%; *P* < 0.001), with a numerically higher adherence rate of recommended 60 mg (1790; 83.8%) than recommended 30 mg (1,479; 71.9%) (Table 1).

The baseline demographics and clinical characteristics of the patients based on the dosing recommendation in accordance with label, with 1790 patients (36.7%) initially received recommended 60 mg edoxaban, 578 (11.9%) received non-recommended 30 mg, 1,479 patients (30.3%) received recommended 30 mg, and 345 patients (7.1%) received non-recommended 60 mg, are presented in Table 2.

**TABLE 2:** Baseline demographics and clinical characteristics by dosing recommendation in accordance with label*.

Baseline characteristics	Recommended 60 mg [n = 1790] (36.7%)	Non-recommended 30 mg [n = 578] (11.9%)	P value	Recommended 30 mg [n = 1,479] (30.3%)	Non-recommended 60 mg [n = 345] (7.1%)	P value
Male, n (%)	1,278 (71.4%)	371 (64.2%)	0.001	506 (34.2%)	161 (46.7%)	<0.001
Age (years), mean (SD)	67.0 (8.9)	71.4 (9.1)	<0.001	74.2 (8.8)	71.5 (8.6)	<0.001
By age sub-groups, n (%)	​	​	<0.001	​	​	<0.001
<65 years	585 (32.7%)	109 (18.9%)	-	184 (12.4%)	64 (18.6%)	-
65–74 years	854 (47.7%)	243 (42.0%)	-	512 (34.6%)	150 (43.5%)	-
75–84 years	333 (18.6%)	194 (33.6%)	-	643 (43.5%)	112 (32.5%)	-
≥85 years	18 (1.0%)	32 (5.5%)	-	140 (9.5%)	19 (5.5%)	-
Body weight^b^ (kg), mean (SD)	75.3 (10.1)	72.8 (8.4)	<0.001	56.1 (7.9)	60.5 (6.8)	<0.001
BMI^c^ (kg/m^2^), mean (SD)	26.7 (3.2)	26.3 (3.1)	0.004	22.1 (2.8)	23.2 (2.6)	<0.001
By BMI subgroups, n (%)	​	​	0.03	​	​	<0.001
<18.5 kg/m^2^	0 (0.0%)	0 (0.0%)	-	124 (8.4%)	6 (1.7%)	-
(18.5–25) kg/m^2^	553 (30.9%)	205 (35.5%)	-	1,122 (75.9%)	265 (76.8%)	-
≥25 kg/m^2^	1,229 (68.7%)	363 (62.8%)	-	202 (13.7%)	70 (20.3%)	-
(calc) creatinine clearance^d^ (mL/min), mean (SD)	85.4 (25.2)	76.4 (21.5)	<0.001	51.3 (19.5)	57.5 (19.1)	<0.001
By CrCL (mL/min), n (%)	​	​	<0.001	​	​	0.002
≥80 mL/min	920 (51.4%)	201 (34.8%)	-	96 (6.5%)	34 (9.9%)	-
≥50–<80 mL/min	870 (48.6%)	377 (65.2%)	-	477 (32.3%)	126 (36.5%)	-
≥30–<50 mL/min	0 (0.0%)	0 (0.0%)	-	586 (39.6%)	123 (35.7%)	-
<30 mL/min	0 (0.0%)	0 (0.0%)	-	121 (8.2%)	10 (2.9%)	-
(calc) eGFR^e^ (mL/min/1.73 m^2^), mean (SD)	66.9 (17.8)	61.2 (16.2)	<0.001	47.6 (18.3)	51.3 (17.9)	0.001
(calc) CHA_2_DS_2_-VASc score^f^, mean (SD)	2.5 (1.3)	3.0 (1.4)	<0.001	3.4 (1.4)	3.1 (1.3)	<0.001
(calc) CHA_2_DS_2_-VA score^g^, mean (SD)	2.7 (1.4)	3.1 (1.5)	<0.001	3.2 (1.5)	2.9 (1.4)	0.01
(mod) HAS-BLED score^h^, mean (SD)	1.5 (1.0)	1.9 (1.0)	<0.001	2.1 (0.9)	2.0 (0.9)	0.04
DOAC score^i^, mean (SD)	3.5 (2.1)	4.4 (2.2)	<0.001	5.4 (2.3)	4.7 (2.3)	<0.001
Type of AF^j^, *n* (%)	​	​	0.51	​	​	0.11
Paroxysmal	660 (36.9%)	217 (37.5%)	-	575 (38.9%)	117 (33.9%)	-
Persistent	585 (32.7%)	200 (34.6%)	-	478 (32.3%)	107 (31.0%)	-
Permanent	241 (13.5%)	72 (12.5%)	-	194 (13.1%)	49 (14.2%)	-
Long-standing persistent	300 (16.8%)	86 (14.9%)	-	228 (15.4%)	70 (20.3%)	-
LVEF categorised by 40%^k^	​	​	0.27	​	​	0.22
<40%	61 (3.4%)	24 (4.2%)	-	58 (3.9%)	8 (2.3%)	-
≥40%	950 (53.1%)	323 (55.9%)	-	717 (48.5%)	180 (52.2%)	-
Perceived frailty, n (%)	50 (2.8%)	58 (10.0%)	<0.001	151 (10.2%)	25 (7.2%)	0.11
COPD^l^, n (%)	75 (4.2%)	35 (6.1%)	0.08	81 (5.5%)	25 (7.2%)	0.26
Hypertension^m^, n (%)	1,362 (76.1%)	428 (74.0%)	0.35	1,019 (68.9%)	261 (75.7%)	0.02
Heart failure^n^, n (%)	251 (14.0%)	115 (19.9%)	<0.001	255 (17.2%)	41 (11.9%)	0.02
Diabetes mellitus^o^, n (%)	506 (28.3%)	147 (25.4%)	0.20	355 (24.0%)	97 (28.1%)	0.13
Dyslipidaemia^p^, n (%)	498 (27.8%)	124 (21.5%)	0.003	337 (22.8%)	96 (27.8%)	0.06
Valvular disease^q^, n (%)	85 (4.7%)	32 (5.5%)	0.52	108 (7.3%)	23 (6.7%)	0.77
History of ischaemic stroke, n (%)	124 (6.9%)	59 (10.2%)	0.01	111 (7.5%)	22 (6.4%)	0.54
History of TIA, n (%)	43 (2.4%)	18 (3.1%)	0.43	29 (2.0%)	10 (2.9%)	0.38
History of major or CRNM bleeding, n (%)	25 (1.4%)	7 (1.2%)	0.90	19 (1.3%)	1 (0.3%)	0.15
History of major bleeding, n (%)	23 (1.3%)	5 (0.9%)	0.55	16 (1.1%)	1 (0.3%)	0.22
Economic region, n (%)	​	​	<0.001	​	​	0.03
East	960 (53.6%)	217 (37.5%)	-	683 (46.2%)	190 (55.1%)	-
Middle	530 (29.6%)	249 (43.1%)	-	563 (38.1%)	107 (31.0%)	-
Northeast	181 (10.1%)	60 (10.4%)	-	80 (5.4%)	18 (5.2%)	-
West	119 (6.7%)	52 (9.0%)	-	153 (10.3%)	30 (8.7%)	-

^a^ Based on judgeable patients (N = 4,192), not including 685 patients with non-judgable dosing.

^b^ Body weight was missing for six patients.

^c^ BMI, was missing for 53 patients.

^d^ The CrCl calculation was based on the CG, formula and was missing for 251 patients.

^e^ eGFR, was calculated by the Du Bois and Du Boi method; it was missing for 300 patients.

^f^ Derived variables of the CHA_2_DS_2_-VASc, score, CHA_2_DS_2_-VA, score and HAS-BLED, score. 113 patients had a missing CHA_2_DS_2_-VASc, score.

^g^ 134 patients had a missing CHA_2_DS_2_-VA, score.

^h^ The HAS-BLED, score was calculated without ‘labile INR’; the renal impairment item was derived instead of investigator reported; alcohol abuse was defined as >1 drink/d; missing for 246 patients.

^i^ DOAC, score was missing for 282 patients.

^j^ AF, type was missing for 13 patients.

^k^ LVEF, data were missing for 1871 patients. ^k^Perceived frailty was missing for 714 patients.

^l^ COPD, was missing for 70 patients.

^m^ Hypertension was unknown in 14 patients.

^n^ Patients were considered as heart failure with documented congestive HF, documented ischaemic cardiomyopathy, LVEF <40%, or frequent dyspnoea (≥1/d) without COPD, and at least one of the following: documented severe valvular heart disease, documented CAD, post-myocardial infarction, valve replacement or documented hypertension treated with at least three drugs.

^o^ Diabetes mellitus was unknown for 24 patients.

^p^ Dyslipidaemia was unknown for 88 patients.

^q^ Valvular disease was unknown for 90 patients. *P* values were calculated by comparing Recommended 60 mg vs. Non-Recommended 30 mg and Recommended 30 mg vs. Non-Recommended 60 mg dose levels.

AF, atrial fibrillation; BMI, body mass index; COPD, chronic obstructive pulmonary disease; CrCl, creatinine clearance; CRNM, clinically relevant non-major; eGFR, estimated glomerular filtration rate; LVEF, left ventricular ejection fraction; TIA, transient ischaemic attack; SD, standard deviation.

When compared with the patients receiving the recommended 60 mg, the patients receiving non-recommended 30 mg edoxaban OD were older (mean 71.4 years vs. 67.0 years), had a higher proportion of patients with perceived frailty (10.0% vs. 2.8%) and HF (19.9% vs. 14.0%), had higher CHA_2_DS_2_-VASc score (3.0 vs. 2.5), CHA_2_DS_2_-VA score (3.1 vs. 2.7), modified HAS-BLED score (1.9 vs. 1.5), DOAC score (4.4 vs. 3.5), and a greater percentage of impaired renal function (CrCl <80 mL/min: 65.2% vs. 48.6%; all *P* < 0.001).

Similarly, patients receiving the recommended 30-mg edoxaban OD at baseline were older (mean 74.2 years vs. 71.5 years; *P* < 0.001), had higher CHA_2_DS_2_-VASc score (3.4 vs. 3.1; *P* < 0.001), DOAC score (5.4 vs. 4.7; *P* < 0.001), and a greater percentage of impaired renal function (CrCl <80 mL/min: 80.1% vs. 75.1%; *P* = 0.002) than those receiving the non-recommended 60 mg (Table 2).

### 3.2 Dosing adjustment and persistence pattern

Overall, a majority of patients (60 mg: 1,938 [73.1%], 30 mg: 1,626 [73.0%]) completed the 1-year follow-up while continuing to use edoxaban (Table 3; Supplementary Figure S3). A lesser proportion of patients treated with edoxaban 30 mg OD initially at baseline switched to 60 mg OD (32; 1.4%) than from 60 mg at baseline to 30 mg OD (158; 6.0%).

**TABLE 3:** Dosing adjustments in edoxaban-treated patients with AF during a 1-year follow-up period.

Dosing adjustment pattern	Total [*n* = 4,877]^a^	60 mg [*n* = 2,650]	30 mg [*n* = 2,227]
Continuing edoxaban use^b^	3,564 (73.1%)	1,938 (73.1%)	1,626 (73.0%)
Dosing adjustments	190 (3.9%)	​	​
Switching to 30 mg OD^c^	-	158 (6.0%)	-
Switching to 60 mg OD^c^	-	-	32 (1.4%)
Persistence pattern			
Study drug suspension^d^	495 (10.1%)	277 (10.5%)	218 (9.8%)
Discontinuation from study drug^e^	777 (15.9%)	380 (14.3%)	397 (17.8%)
Immediate switching to other OAC^f^			
Warfarin	32 (0.7%)	16 (0.6%)	16 (0.7%)
Apixaban	1 (0.02%)	-	1 (0.04%)
Dabigatran	13 (0.3%)	9 (0.3%)	4 (0.2%)
Rivaroxaban	47 (1.0%)	23 (0.9%)	24 (1.1%)
Suspension and discontinuation from study drug^g^	76 (1.6%)	37 (1.4%)	39 (1.8%)

^a^ Based on full analysis set (FAS).

^b^ Continued edoxaban use: continuously using edoxaban during 1-year follow-up however allowing for interruptions ≤3 days and for a switch of dose <90 days before the 1 year cut-off.

^c^ Switching to 30 mg or 60 mg: switch to another dose of edoxaban after baseline with a duration of ≥90 days or until 1-year follow-up.

^d^ Study drug suspension: stopped edoxaban for >3 days and restarted in ≤90 days.

^e^ Discontinuation from study drug: stopped edoxaban for >3 days, not continued in ≤90 days at the end of 1-year follow-up.

^f^ Immediate switching to other OAC: discontinuation from edoxaban, switch to other OAC, in ≤7 days.

^g^ Suspend and discontinuation from study drug: stopped edoxaban for >3 days, restarted edoxaban in ≤90 days, but discontinued at the end of 1-year follow-up.

OAC, oral anticoagulants; OD, once daily.

In the 60 mg group, 277 (10.5%) patients suspended edoxaban and 380 patients (14.3%) discontinued the drug during 1-year follow-up. Similarly, in the 30 mg group, edoxaban was suspended in 218 patients (9.8%), whereas 397 patients (17.8%) discontinued. Within each group, the percentages of patients who discontinued edoxaban and immediately switched to another OAC were less than or ∼1% (60 mg: warfarin 16 [0.6%], dabigatran 9 [0.3%] and rivaroxaban 23 [0.9%]; 30 mg: warfarin 16 [0.7%], apixaban 1 [0.04%], dabigatran 4 [0.2%] and rivaroxaban 24 [1.1%]). Suspension or discontinuation of edoxaban was observed in 37 patients (1.4%) in the 60 mg group and in 39 patients (1.8%) in the 30 mg group.

### 3.3 Mortality, bleeding, and CV clinical outcomes

**FIGURE 1:**
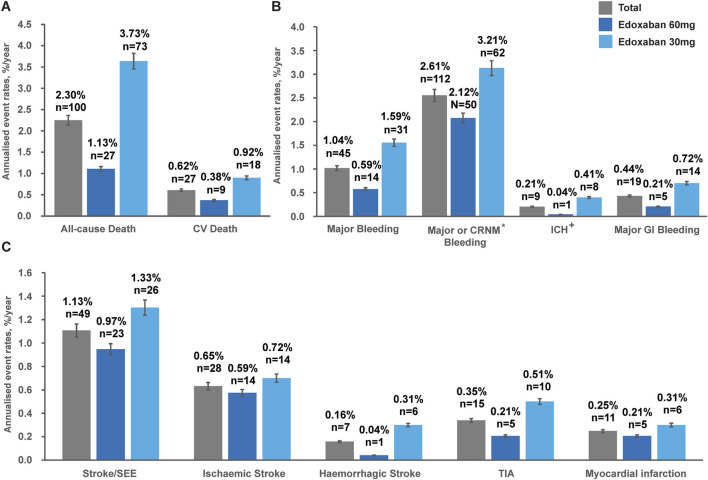
Annualised event rates^#^ (%/y) of: **(A)** Death outcomes, **(B)** Safety outcomes and **(C)** Effectiveness outcomes in the overall population^*^ classified by edoxaban dose at baseline during the 1-year follow-up. *Based on full analysis set (FAS), N = 4,877. #Patients with no official clinic visit date during 12 months of follow-up were censored at last-available events or hospitalisations or AEs record date. ^+^Included haemorrhagic stroke or epidural/subdural haematoma. CV, cardiovascular; CRNM, clinically relevant non-major; ICH, intracranial haemorrhage; GI, gastrointestinal; SEE, systemic embolic event; TIA, transient ischaemic attack.

The overall annualised event rate of all-cause death was 2.30%/y in the total population (Figure 1). The overall annualised rates were low for CV death (0.62%/y), major bleeding (1.04%/y), ICH (0.21%/y), and major GI bleeding (0.44%/y). Stroke/SEE occurred in 49 patients (1.13%/y), of whom 28 patients (0.65%/y) had ischaemic stroke and seven patients (0.16%/y) had haemorrhagic stroke. The incidences of TIA and MI were 0.35%/y and 0.25%/y, respectively, in the overall patient population.

**FIGURE 2:**
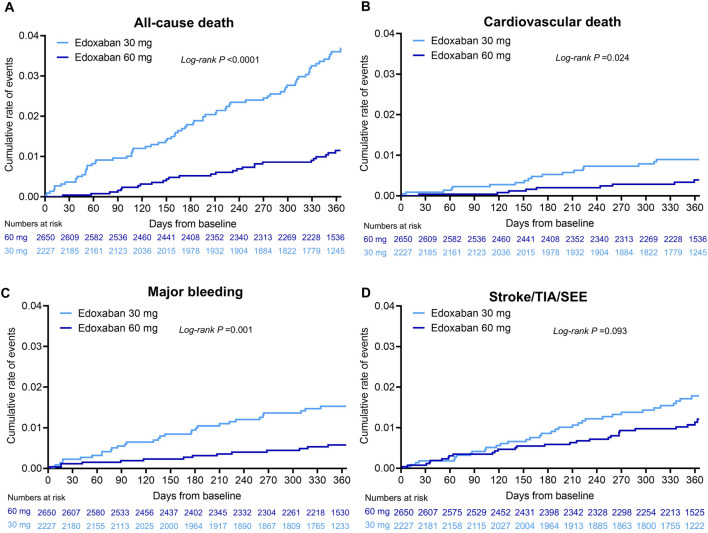
Cumulative incidence for **(A)** all-cause death, **(B)** cardiovascular death, **(C)** major bleeding, **(D)** stroke/SEE/TIA in patients^*^ with AF at 1-year follow-up classified by edoxaban dose. *Based on full analysis set (FAS), N = 4,877. *P* value was calculated by log-rank test. AF, atrial fibrillation; SEE, systemic embolic event; TIA, transient ischaemic attack.

Unadjusted annualised event rates, which should be interpreted with caution due to significant baseline differences between dose groups (Table 1), showed that during the 1-year follow-up period, the overall annualised rates of all-cause death in the 30 mg and 60 mg edoxaban remained constant over time (Figure 2A). Patients receiving 30 mg edoxaban had a higher risk of all-cause death as well as CV death when compared with patients receiving 60 mg edoxaban (all-cause death: 3.73%/y vs. 1.13%/y, log-rank P<0.0001; CV deaths: 0.92%/y vs. 0.38%/y, *P* = 0.024; Figures 2A,B). A larger proportion of patients in the 30 mg edoxaban group than 60 mg edoxaban group experienced major bleeding (1.59%/y vs. 0.59%/y, *P* = 0.001; Figure 2C). No significant differences in the annualised rates of stroke/SEE, or TIA between the dose groups were observed (Figure 2D; *P* > 0.05).

### 3.4 Predictors and outcomes by dosing groups

**FIGURE 3:**
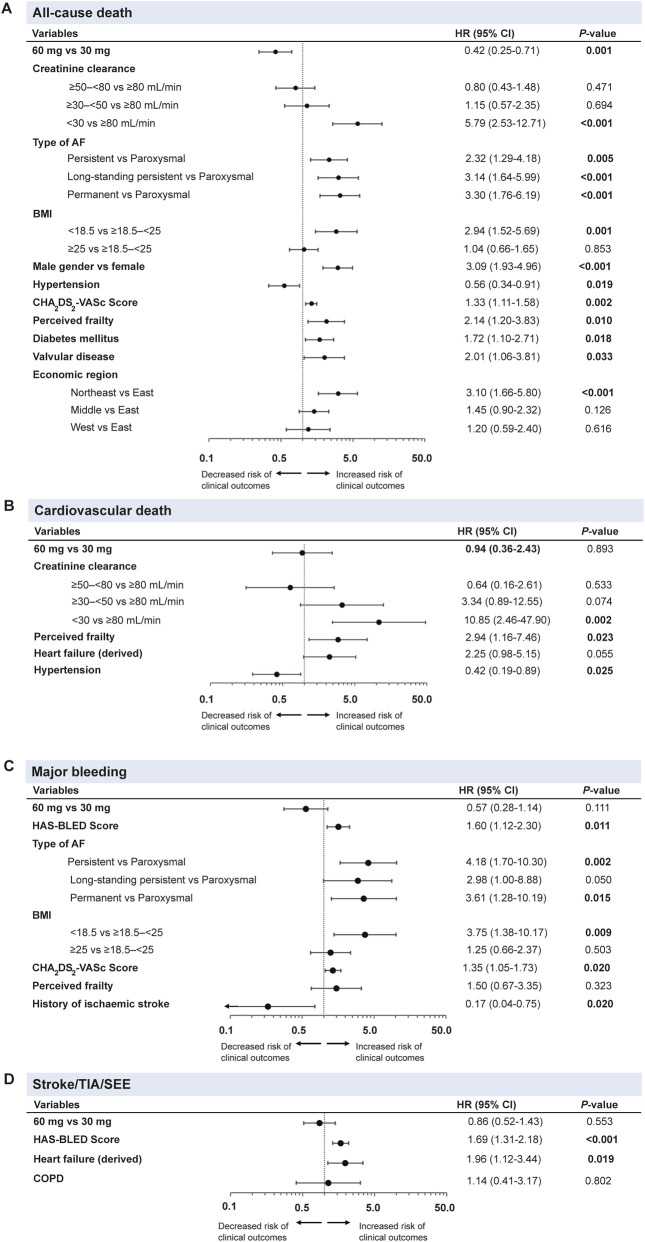
Multivariable cox regression analysis^#^ of dosing and characteristics associated with **(A)** all-cause death, **(B)** cardiovascular death, **(C)** major bleeding and **(D)** stroke/TIA/SEE outcomes of overall population^*^ during the 1-year follow-up. #Multivariable cox regression analysis by fixing edoxaban dosing (60 mg and 30 mg) and all characteristics in a stepwise regression model. *Based on full analysis set (FAS), N = 4,877. Forest plots represented data as HRs with a 95% CI. AF, atrial fibrillation; BMI, body mass index; COPD, chronic obstructive pulmonary disease; CI, confidence interval; HR, hazard ratio; SEE, systemic embolic event; TIA, transient ischaemic attack.

The factors associated with all-cause death, CV death, all stroke/SEE/TIA, and major bleeding were significant if *P* < 0.05 of univariable analysis (Supplementary Tables S2–S5). The selected factors were further adjusted by fixing edoxaban dosing (60 and 30 mg) in a stepwise multivariable Cox regression model (Figure 3). A CrCl <30 mL/min (HR: 5.79, 95% CI: 2.53–12.71; P<0.0001), underweight (BMI < 18.5; HR: 2.94, 95% CI: 1.52–5.69; *P* = 0.001), CHA_2_DS_2_-VASc scores (HR: 1.33, 95% CI: 1.11–1.58; P = 0.002), long-standing persistent AF (HR: 3.14, 95% CI: 1.64–5.99; P<0.0001) and permanent AF (HR: 3.30, 95% CI: 1.76–6.19; *P*<0.0001) were the main clinically significant factors related to all-cause death (Figure 3A). Male gender (HR: 3.09, 95% CI: 1.93–4.96; P<0.0001), perceived frailty (HR: 2.14, 95% CI: 1.20–3.83; P<0.0001), and economic regions of Northeast (HR: 3.10, 95% CI: 1.66–5.80; P<0.0001) were further identified as the predictors of all-cause death. Similarly, CrCl <30 mL/min (HR: 10.85, 95% CI: 2.46–47.90; *P* = 0.002) and perceived frailty (HR: 2.94, 95% CI: 1.16–7.46; *P* = 0.023) were also associated with CV death (Figure 3B). The persistent AF type (HR: 4.18, 95% CI: 1.70–10.30; *P* = 0.002) was a strong predictor of major bleeding events, in addition to permanent AF (HR: 3.61, 95% CI: 1.28–10.19; *P* = 0.015), BMI of <18.5 (HR: 3.75, 95% CI: 1.38–10.17; *P* = 0.009), HAS-BLED scores (HR: 1.60, 95% CI: 1.12–2.30; *P* = 0.011) and CHA_2_DS_2_-VASc scores (HR: 1.35, 95% CI: 1.05–1.73; *P* = 0.020) (Figure 3C). For stroke/SEE/TIA, HAS-BLED score (HR: 1.69, 95% CI: 1.31–2.18; *P*<0.0001) and HF (HR: 1.96, 95% CI: 1.12–3.44; *P* = 0.019) were relevant predictors (Figure 3D).

Upon performing the step-wise multivariable cox regression analysis of recommended/non-recommended doses, patients on non-recommended doses (both under- and over-dosing) often had worse outcomes, likely reflecting their higher baseline risk profile rather than a direct effect of the dosing itself (Supplementary Table S6).

### 3.5 Outcomes in the recommended versus non-recommended dose subgroups

After 1-year follow-up, patients who were prescribed the non-recommended 30 mg dose of edoxaban had higher risks of all-cause mortality (HR: 2.49, 95% CI: 1.27–4.91; *P* = 0.006), major bleeding (HR: 3.54, 95% CI: 1.37–9.17, *P* = 0.005), and major GI bleeding (HR: 5.22, 95% CI: 1.25–21.85, *P* = 0.01) compared with the recommended 60 mg dose (Supplementary Figure S4). Patients receiving the non-recommended 60 mg versus the recommended 30 mg had lower annualised event rates of all-cause mortality (HR: 0.40, 95% CI: 0.16–0.99; *P* = 0.04). The rates of stroke/SEE/TIA and ischaemic stroke were similar between non-recommended 30 mg versus recommended 60 mg or non-recommended 60 mg versus recommended 30-mg dose groups (all *P* > 0.05).

## 4 Discussion

The 1-year follow-up data from the ETNA-AF China study initially reported the effectiveness and safety of edoxaban in a large Chinese population with AF. The results demonstrated low rates of all stroke/SEE (1.13%/y) and major bleeding events (1.04%/y) in consistency with the international, prospective, and noninterventional Global ETNA-AF programme, complementing the pivotal trials by encompassing the non-selected Chinese a population in real-world setting. This report also highlights various predictors of all-cause and CV death, major bleeding, and stroke events in patients treated with recommended/non-recommended 30 mg and 60 mg doses of edoxaban, indicating integrated AF care through risk factor modification.

The current results from China though not yet pooled with the full global database highlight the similarities as well as regional differences between other ETNA studies. At the 1-year follow-up, patients from Europe, Japan, the Chinese Mainland, South Korea, and Taiwan (China) displayed similar annualised rates of stroke or SEE (0.82%, 1.43%, 1.13%, 1.27% [any stroke], 1.52% [any stroke]) and major bleeding events (1.05%, 1.08%, 1.04%, 0.99%, 1.37%) with analogous CHA_2_DS_2_-VASc and HAS-BLED score categories (Yamashita et al., 2020; Choi et al., 2021; Choi et al., 2024; De Groot et al., 2021). The annualised all-cause and CV death rates were notably lower in our study (2.30% and 0.62%, respectively) compared with the 1-year outcome of the ETNA-AF-Europe study (3.50% and 1.63%, respectively) (De Groot et al., 2021). The younger age (70.3 years vs. 73.6 years), lower average body weight (67.2 kg vs. 81.0 kg) and lower perceived frailty (6.3% vs. 10.6%) characteristics could explain the lower mortality events in the Chinese population compared with the European population. ETNA-AF Japan and ETNA-AF Other Asia studies reported slightly lower 1-year interim all-cause death rates (1.22% and 1.78%, respectively) (Yamashita et al., 2020). Patients from Japan had the highest adherence rate on label-recommended doses of 85.2% among all ETNA-AF programmes (Europe: 83%; Chinese Mainland: 78.0%; South Korea/Taiwan: 70.8%), which may contribute to the better survival outcomes (Chao et al., 2023). On one hand, the 1-year interim annualised rates of major or CRNM bleeding were also observed to be the highest in ETNA-AF Japan (Japan: 3.83%, Europe: 2.35%, Chinese Mainland: 2.54%, and Other Asia: 2.18%). On the other hand, ETNA-AF Other Asia reported the highest rates of ischaemic stroke, haemorrhagic stroke, major bleeding, ICH, as well as major GI bleeding. This may be attributed to Asian patients having a higher bleeding risk and lower body weight than other populations (Giugliano et al., 2013; Chan et al., 2016; Cha et al., 2017; Choi et al., 2024).

A comparison of outcomes between two dose groups revealed a higher risk of all-cause death, CV death, and major bleeding in patients receiving edoxaban 30 mg OD than those on 60 mg OD. This may be overwhelmingly attributed to the poorer health status of 30 mg edoxaban group, who were generally older (mean age: 73.4 vs. 67.6 years), had higher CHA_2_DS_2_-VASc (mean: 3.3 vs. 2.6) and CHA_2_DS_2_-VA scores (mean: 3.1 vs. 2.6), had higher DOAC score (mean: 5.1 vs. 3.6), had a greater prevalence of frailty (9.9% vs. 3.4%) and had further impaired renal function (CrCl <80 mL/min, 70.5% vs. 42.8%). These 1-year interim results are consistent with that of the 1-year results of ETNA-AF Europe and Japan (Yamashita et al., 2020; De Groot et al., 2021).

The annualised rates of stroke/SEE and major bleeding in our study were relatively low compared with other real-world registries of Asian patients (Supplementary Table S7). At 1-year follow-up, the rates of stroke/SEE and major bleeding were lower in ETNA-AF-China in comparison with the XANAP and XAPASS studies (Kim et al., 2018; Ikeda et al., 2019). In addition, our findings were consistent with the GLORIA-AF Asian cohort, in which the incidences of all strokes and major bleeding were both 0.90/100 PY after 2-year dabigatran treatment (HAS-BLED score of 1.1) (Mazurek et al., 2019). Notably, comparing event rates between 1-year and 2-year treatments shows variations, with lower rates being observed in the second year. The 1-year event rates of stroke/SEE and major bleeding of Chinese patients using edoxaban were on par with NOACs in Asian or global patients (Okumura et al., 2018; Bassand et al., 2019).

The exploration of CV risk factors for outcomes of mortality, bleeding and stroke may help enlighten the clinical management of AF (Kirchhof et al., 2022). Renal impairment categorised by CrCl value was identified as a significant dose-adjusted factor for all-cause death (CrCl <30 mL/min: HR: 5.79, 95% CI: 2.53–12.71; *P* < 0.001), and CV death HR: 10.85, 95% CI: 2.46–47.90; *P* = 0.002). Furthermore, the type of AF, specifically permanent and persistent AF (persistent AF vs. paroxysmal AF, HR: 4.18, 95% CI: 1.70–10.30; *P* = 0.002), BMI (BMI <18.5 vs. BMI ≥18.5–<25, HR: 3.75, 95% CI: 1.38–10.17; *P* = 0.009), and baseline CHA_2_DS_2_-VASc score (HR: 1.35, 95% CI: 1.05–1.73; *P* = 0.020) were also identified as significant dose-adjusted risk factors for major bleeding and all-cause death ([long-standing persistent AF vs. paroxysmal AF] HR: 3.14, 95% CI: 1.64–5.99; P < 0.001; [persistent AF vs. paroxysmal AF]; HR: 3.30, 95% CI: 1.76–6.19; *P* < 0.001; [BMI <18.5 vs. BMI ≥18.5–<25]; HR: 2.94, 95% CI: 1.52–5.69; *P* = 0.001; [CHA_2_DS_2_-VASc score] HR: 1.33, 95% CI: 1.11–1.58; *P* = 0.002). On par with our study, the ENGAGE AF-TIMI 48 trial showed higher incidences of all-cause death and major adverse cardiovascular events (MACE) in persistent and permanent AF (Link et al., 2017). However, in the sub-group analysis of the ENTRUST-AF PCI trial and ENSURE-AF study, paroxysmal AF was associated with a higher occurrence of MI (Goette et al., 2020; Goette et al., 2021). The EAST-AFNET four trial reported that patients with first-diagnosed AF were at a higher risk of hospitalization because of acute coronary syndrome (ACS; incidence rate ratio: 1.50; 95% CI: 0.83–2.69) when compared with paroxysmal and persistent AF (Goette et al., 2022). In the present study, patients with different AF type reported no significant difference on 1-year event rate of ACS (*P* > 0.05), whereas those with permanent AF (4 [0.68%]) and paroxysmal AF (5 [0.32%]) reported numerically the highest rate of MI (*P* = 0.08) (Supplementary Figure S5). This may be attributed to the small ASC event (including MI) amount occurred during 1-year follow-up. Perceived frailty was also reported as a predictor for all-cause death (HR: 2.14, 95% CI: 1.20–3.83; *P* = 0.010) and CV death (HR: 2.94, 95% CI: 1.16–7.46; *P* = 0.023), respectively. Furthermore, HF (HR: 1.96, 95% CI: 1.12–3.44; *P* = 0.019) was identified as a factor for stroke/SEE/TIA. This focus from the ETNA-AF-China study provides valuable insights into the nuanced factors that influence the outcomes in patients with AF, warranting a further careful assessment of the stroke and bleeding risks.

Overall, worse clinical benefits were recognised in the non-recommended low dose by exploratory analysis. It was found that the non-recommended 30 mg was associated with a higher risk of all-cause death (HR: 2.43, 95% CI: 1.222–4.82; *P* = 0.011) and major bleeding (HR: 2.90, 95% CI: 1.10–7.66; *P* = 0.032) vs. recommended 60 mg edoxaban in multivariable cox regression model. However, residual confounding is likely and the observed results may be attributed to the baseline characteristics of this group representing the ““sicker”“ patients, such as lower body weight, renal impairment, advanced age, higher CHA_2_DS_2_-VASc scores, and higher modified HAS-BLED scores, where the non-recommended dose is a marker of severity, not necessarily the cause of the poor outcome. Long-term monitoring is needed to better understand the risks and benefits of non-recommended dosing, particularly for higher doses in Asian patients. Studies have also shown different outcomes in recommended vs. non-recommended doses of NOACs based on regional and ethnic differences, underscoring the importance of individualized dose selection and patient-tailored decision-making to optimize clinical outcomes and minimize bleeding risk (Chan et al., 2023; Lip et al., 2023).

### 4.1 Strengths and limitations

The strengths of the ETNA-AF-China study were the inclusion of 4,877 patients and the presentation of comprehensive 1-year follow-up data by interim cut-off, which provided significant insights into the demographic, clinical characteristics, safety, and effectiveness outcomes of patients with AF prescribed edoxaban at the beginning of the study. This study fully collected dose reduction criteria of edoxaban (including renal function), which can provide information for judgment of recommended and non-recommended dosing regimens of NOACs in clinical management of AF. The reflection of real-world conditions, including the extensive sample size, its prospective study design, and the autonomy of physicians in making therapeutic choices, enhances the reliability and applicability of the gathered data in the ETNA-AF-China study.

This study, however, has certain limitations. Owing to its observational and open-label design, detailed data on laboratory tests and other assessments were not collected. The single-arm study design only reported the effectiveness and safety of edoxaban, not a comparison or control against other anticoagulants such as NOAC or VKA. In addition, patients were assigned to the 30 mg or 60 mg edoxaban dose based on clinical characteristics rather than randomization, making direct comparisons between dose groups potentially confounded by indication and underlying health differences rather than the drug’s effect alone. Therefore, the reported *P* values should be interpreted in an exploratory or descriptive manner, since no adjustment for multiple testing was made. The subgroup analyses are further limited by their small sample size, multiple testing, and potential instability of the model due to small event numbers. Moreover, the annualised event rates were estimated by censoring those lost to follow-up or premature termination (11.2%), which may lead to underreported events or co-medications. As this is a real-world, observational study, there was no mandatory requirement for participants or investigators to complete follow-up. This inherent lack of compulsion may have led to incomplete data capture. Furthermore, the current data of ETNA-AF China are not mature enough to be pooled with the global ETNA-AF database, as analyses are still undergoing alignment to standardize results across ETNA-AF studies.

## 5 Conclusion

Routine treatment using edoxaban was found to be effective and safe across a broad spectrum of Chinese patients, with low overall annualised incidence rates of CV mortality, major bleeding (notably ICH), and major GI bleeding during the 1-year follow-up period. Edoxaban dosing (30 mg/60 mg, recommended/non-recommended doses), type of AF, BMI and baseline comorbidities (frailty, diabetes) were identified as potential risk factors of all-cause death and major bleeding, with edoxaban 30 mg dose being appropriately prescribed to a higher-risk population in whom it appeared to have an acceptable risk-benefit profile. At the end of the 1-year follow-up, the majority of patients continued routinely using edoxaban.

## Data Availability

The raw data supporting the conclusions of this article will be made available by the authors, complying with local information security protection laws and regulations in China.

## References

[B1] Aggarwal R. Ruff C. T. Virdone S. Perreault S. Kakkar A. K. Palazzolo M. G. (2023). Development and validation of the DOAC score: a novel bleeding risk prediction tool for patients with atrial fibrillation on direct-acting oral anticoagulants. Circulation 148, 936–946. 10.1161/CIRCULATIONAHA.123.064556 37621213 PMC10529708

[B2] Bassand J.-P. Virdone S. Goldhaber S. Z. Camm A. J. Fitzmaurice D. A. Fox K. A. A. (2019). Early risks of death, stroke/systemic embolism, and major bleeding in patients with newly diagnosed atrial fibrillation: results from the GARFIELD-AF registry. Circulation 139, 787–798. 10.1161/CIRCULATIONAHA.118.035012 30586740

[B3] Benjamin E. J. Muntner P. Alonso A. Bittencourt M. S. Callaway C. W. Carson A. P. (2019). Heart disease and stroke Statistics-2019 update: a report from the American heart association. Circulation 139, e56–e528. 10.1161/CIR.0000000000000659 30700139

[B4] Cha M.-J. Choi E.-K. Han K.-D. Lee S.-R. Lim W.-H. Oh S. (2017). Effectiveness and safety of non-vitamin K antagonist oral anticoagulants in Asian patients with atrial fibrillation. Stroke 48, 3040–3048. 10.1161/STROKEAHA.117.018773 28974629

[B5] Chan Y.-H. Kuo C.-T. Yeh Y.-H. Chang S.-H. Wu L.-S. Lee H.-F. (2016). Thromboembolic, bleeding, and mortality risks of rivaroxaban and dabigatran in Asians with nonvalvular atrial fibrillation. J. Am. Coll. Cardiol. 68, 1389–1401. 10.1016/j.jacc.2016.06.062 27659460

[B6] Chan Y.-H. Chan C.-Y. Chen S.-W. Chao T.-F. Lip G. Y. H. (2023). Comparisons of effectiveness and safety between on-label dosing, off-label underdosing, and off-label overdosing in Asian and Non-Asian atrial fibrillation patients treated with rivaroxaban: a systematic review and meta-analysis of observational studies. EP Eur. 25, euad288. 10.1093/europace/euad288 37738425 PMC10580379

[B7] Chao T.-F. Unverdorben M. Kirchhof P. Koretsune Y. Yamashita T. Crozier R. A. (2023). Prescribing patterns and outcomes of edoxaban in atrial fibrillation: one-year data from the global ETNA-AF program. JCM 12, 1870. 10.3390/jcm12051870 36902656 PMC10003604

[B8] Chinese Society of Cardiology, Chinese Medical Association and Heart Rhythm Committee of Chinese Society of Biomedical EngineeringHeart Rhythm Committee of Chinese Society of Biomedical Engineering (2023). Chinese guidelines on diagnosis and management of atrial fibrillation. Chin. J. Cardiol. 51, 572–618. 10.3760/cma.j.cn112148-20230416-00221 37312479

[B9] Choi E.-K. Lin W.-S. Hwang G.-S. Kirchhof P. De Caterina R. Chen C. (2021). Clinical events with edoxaban in South Korean and Taiwanese atrial fibrillation patients in routine clinical practice. J. Clin. Med. 10, 5337. 10.3390/jcm10225337 34830618 PMC8623093

[B10] Choi J.-I. Kiatchoosakun S. Jiampo P. Tse H. F. Soo Y. O. Y. Wang C.-C. (2024). Prescribing patterns and outcomes of edoxaban in atrial fibrillation patients from Asia ― one-year data from the global ETNA-AF program. Circ. Rep. 6, 86–93. 10.1253/circrep.CR-23-0098 38464984 PMC10920013

[B11] De Caterina R. Agnelli G. Laeis P. Unverdorben M. Rauer H. Wang C. (2019a). The global edoxaban treatment in routine cliNical prActice (ETNA) noninterventional study program: rationale and design. Clin. Cardiol. 42, 1147–1154. 10.1002/clc.23279 31650560 PMC6906985

[B12] De Caterina R. Kelly P. Monteiro P. Deharo J. C. de Asmundis C. López-de-Sá E. (2019b). Design and rationale of the edoxaban treatment in routiNe clinical prActice for patients with atrial fibrillation in Europe (ETNA-AF-Europe) study. J. Cardiovasc Med. 20, 97–104. 10.2459/JCM.0000000000000737 30540648

[B13] De Caterina R. Kim Y.-H. Koretsune Y. Wang C.-C. Yamashita T. Chen C. (2021). Safety and effectiveness of edoxaban in atrial fibrillation patients in routine clinical practice: one-year Follow-Up from the global noninterventional ETNA-AF program. JCM 10, 573. 10.3390/jcm10040573 33546442 PMC7913627

[B14] De Groot J. R. Weiss T. W. Kelly P. Monteiro P. Deharo J. C. De Asmundis C. (2021). Edoxaban for stroke prevention in atrial fibrillation in routine clinical care: 1-Year follow-up of the prospective observational ETNA-AF-Europe study. Eur. Heart J. 7, f30–f39. 10.1093/ehjcvp/pvaa079 32790837 PMC8117428

[B15] European Medicines Agency (2024). Lixiana | European medicines agency. Available online at: https://www.ema.europa.eu/en/medicines/human/EPAR/lixiana (Accessed May 6, 2024).

[B16] Feigin V. L. Brainin M. Norrving B. Martins S. O. Pandian J. Lindsay P. (2025). World stroke organization: global stroke fact sheet 2025. Int. J. Stroke 20, 132–144. 10.1177/17474930241308142 39635884 PMC11786524

[B17] Giugliano R. P. Ruff C. T. Braunwald E. Murphy S. A. Wiviott S. D. Halperin J. L. (2013). Edoxaban *versus* warfarin in patients with atrial fibrillation. N. Engl. J. Med. 369, 2093–2104. 10.1056/NEJMoa1310907 24251359

[B18] Goette A. Lip G. Y. H. Jin J. Heidbuchel H. Cohen A.-A. Ezekowitz M. (2020). Differences in thromboembolic complications between paroxysmal and persistent atrial fibrillation patients following electrical cardioversion (from the ENSURE-AF study). Am. J. Cardiol. 131, 27–32. 10.1016/j.amjcard.2020.06.046 32753268

[B19] Goette A. Eckardt L. Valgimigli M. Lewalter T. Laeis P. Reimitz P.-E. (2021). Clinical risk predictors in atrial fibrillation patients following successful coronary stenting: ENTRUST-AF PCI sub-analysis. Clin. Res. Cardiol. 110, 831–840. 10.1007/s00392-020-01760-4 33098470 PMC8166657

[B20] Goette A. Borof K. Breithardt G. Camm A. J. Crijns H. J. G. M. Kuck K.-H. (2022). Presenting pattern of atrial fibrillation and outcomes of early rhythm control therapy. JACC 80, 283–295. 10.1016/j.jacc.2022.04.058 35863844

[B21] Guo X. Du J. Yang Y. Wu M. Ou W. Han X. (2024). Edoxaban for stroke prevention in atrial fibrillation and factors associated with dosing: patient characteristics from the prospective observational ETNA-AF-China registry. Sci. Rep. 14, 2778. 10.1038/s41598-024-51776-3 38307927 PMC10837439

[B22] Hindricks G. Potpara T. Dagres N. Arbelo E. Bax J. J. Blomström-Lundqvist C. (2021). 2020 ESC guidelines for the diagnosis and management of atrial fibrillation developed in collaboration with the European association for cardio-thoracic surgery (EACTS). Eur. Heart J. 42, 373–498. 10.1093/eurheartj/ehaa612 32860505

[B23] Ikeda T. Ogawa S. Kitazono T. Nakagawara J. Minematsu K. Miyamoto S. (2019). Real-world outcomes of the Xarelto post-authorization safety and effectiveness study in Japanese patients with atrial fibrillation (XAPASS). J. Cardiol. 74, 60–66. 10.1016/j.jjcc.2019.01.001 30745002

[B24] Jiang J. Weng Y. Huang J. Deng H. Liao H. Fang X. (2024). Current anticoagulation statuses among older Chinese people with nonvalvular atrial fibrillation. Rev. Cardiovasc Med. 25, 79. 10.31083/j.rcm2503079 39076934 PMC11263859

[B25] Joglar J. A. Chung M. K. Armbruster A. L. Benjamin E. J. Chyou J. Y. Cronin E. M. (2024). 2023 ACC/AHA/ACCP/HRS guideline for the diagnosis and management of atrial fibrillation: a report of the American college of cardiology/American heart association joint committee on clinical practice guidelines. Circ 149, e1–e156. 10.1161/CIR.0000000000001193 38033089 PMC11095842

[B26] Kim Y. Shim J. Tsai C. Wang C. Vilela G. Muengtaweepongsa S. (2018). XANAP: a real‐world, prospective, observational study of patients treated with rivaroxaban for stroke prevention in atrial fibrillation in Asia. J. Arrhythm. 34, 418–427. 10.1002/joa3.12073 30167013 PMC6111488

[B27] Kirchhof P. Pecen L. Bakhai A. De Asmundis C. De Groot J. R. Deharo J. C. (2022). Edoxaban for stroke prevention in atrial fibrillation and age-adjusted predictors of clinical outcomes in routine clinical care. Eur. Heart J. 9, 47–57. 10.1093/ehjcvp/pvac042 35881467 PMC9753092

[B28] Link M. S. Giugliano R. P. Ruff C. T. Scirica B. M. Huikuri H. Oto A. (2017). Stroke and mortality risk in patients with various patterns of atrial fibrillation: results from the ENGAGE AF-TIMI 48 trial (effective anticoagulation with factor Xa next generation in atrial fibrillation–thrombolysis in myocardial infarction 48). Circ Arrhythmia Electrophysiol. 10, e004267. 10.1161/CIRCEP.116.004267 28077507

[B29] Lip G. Y. H. Proietti M. Potpara T. Mansour M. Savelieva I. Tse H. F. (2023). Atrial fibrillation and stroke prevention: 25 years of research at EP Europace journal. Europace 25, euad226. 10.1093/europace/euad226 37622590 PMC10451006

[B30] Lippi G. Sanchis-Gomar F. Cervellin G. (2021). Global epidemiology of atrial fibrillation: an increasing epidemic and public health challenge. IJS 16, 217–221. 10.1177/1747493019897870 31955707

[B31] Mazurek M. Teutsch C. Diener H.-C. Dubner S. J. Halperin J. L. Ma C.-S. (2019). Safety and effectiveness of dabigatran at 2 years: final outcomes from Phase II of the GLORIA-AF registry program. Am. Heart J. 218, 123–127. 10.1016/j.ahj.2019.08.012 31806087

[B32] Michels W. M. Grootendorst D. C. Verduijn M. Elliott E. G. Dekker F. W. Krediet R. T. (2010). Performance of the cockcroft-gault, MDRD, and new CKD-EPI formulas in relation to GFR, age, and body size. Clin. J. Am. Soc. Nephrol. 5, 1003–1009. 10.2215/CJN.06870909 20299365 PMC2879308

[B33] Okumura Y. Yokoyama K. Matsumoto N. Tachibana E. Kuronuma K. Oiwa K. (2018). Three-year clinical outcomes associated with warfarin vs. direct oral anticoagulant use among Japanese patients with atrial fibrillation ― findings from the SAKURA AF registry. Circ. J. 82, 2500–2509. 10.1253/circj.CJ-18-0535 30078823

[B34] Public Policy Committee, International Society of Pharmacoepidemiology (2016). Guidelines for good pharmacoepidemiology practice (GPP): guidelines for good pharmacoepidemiology practice. Pharmacoepidemiol Drug Saf. 25, 2–10. 10.1002/pds.3891 26537534

[B35] SAVAYSA (2015). SAVAYSA (edoxaban) tablets for oral use initial U.S. approval: 2015. Available online at: https://www.accessdata.fda.gov/drugsatfda_docs/label/2015/206316lbl.pdf.

[B36] Schnabel R. B. Marinelli E. A. Arbelo E. Boriani G. Boveda S. Buckley C. M. (2023). Early diagnosis and better rhythm management to improve outcomes in patients with atrial fibrillation: the 8th AFNET/EHRA consensus conference. EP Eur. 25, 6–27. 10.1093/europace/euac062 35894842 PMC9907557

[B37] Schulman S. Kearon C. Subcommittee on Control of Anticoagulation of the Scientific and Standardization Committee of the International Society on Thrombosis and Haem and ostasis (2005). Definition of major bleeding in clinical investigations of antihemostatic medicinal products in non‐surgical patients. J. Thrombosis Haemostasis 3, 692–694. 10.1111/j.1538-7836.2005.01204.x 15842354

[B38] Shi S. Tang Y. Zhao Q. Yan H. Yu B. Zheng Q. (2022). Prevalence and risk of atrial fibrillation in China: a national cross-sectional epidemiological study. Lancet Regional Health – West. Pac. 23, 100439. 10.1016/j.lanwpc.2022.100439 35800039 PMC9252928

[B39] Sun T. Ye M. Lei F. Qin J.-J. Liu Y.-M. Chen Z. (2023). Prevalence and trend of atrial fibrillation and its associated risk factors among the population from nationwide health check-up centers in China, 2012–2017. Front. Cardiovasc Med. 10, 1151575. 10.3389/fcvm.2023.1151575 37324618 PMC10264614

[B40] Tu W.-J. Wang L.-D. Yan F. Peng B. Hua Y. Liu M. (2023). China stroke surveillance report 2021. Mil. Med. Res. 10, 33. 10.1186/s40779-023-00463-x 37468952 PMC10355019

[B41] Yamashita T. Koretsune Y. Nagao T. Shiosakai K. (2020). Postmarketing surveillance on the clinical use of edoxaban in patients with nonvalvular atrial fibrillation (ETNA‐AF‐Japan): one‐year safety and effectiveness analyses. J. Arrhythm. 36, 395–405. 10.1002/joa3.12332 32528563 PMC7279995

[B42] Zhang C. Wang J. Yang Y. Ma E.-L. Lin H.-W. Liu B.-L. (2022). Prescribing trends of oral anticoagulants from 2010 to 2020 in Shanghai, China: a retrospective study. Clin. Appl. Thromb. Hemost. 28, 107602962211325. 10.1177/10760296221132551 36250531 PMC9578173

