# Supplementary material for: Edoxaban for stroke prevention in Chinese patients with atrial fibrillation: 1-year follow-up of the ETNA-AF-China study
Source: Front Pharmacol. 2026 Jan 22;17:1739246. doi: 10.3389/fphar.2026.1739246 (PMC12872560; doi:10.3389/fphar.2026.1739246)
Supplement: Supplementary file 1 [file DataSheet1.docx]

Supplementary Material

**Supplementary Figure S1.** Flowchart of patients based on the interim cut-off after 1 year of ETNA-AF-China


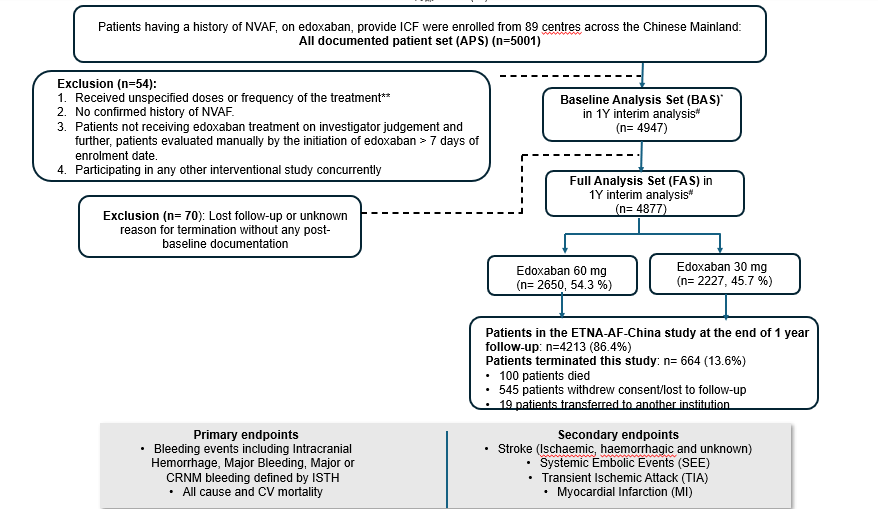


^*^BAS in this 1y-interim analysis refers to all patients from the APS with 1) a confirmed history of NVAF confirmed by electrical tracing (eg. ECG, Holter, Pacemaker, ICD); 2) currently treated with Edoxaban at enrolment/baseline (i.e., ongoing or ≤1 week after baseline); 3) received specified doses/frequency (60 mg/OD or 30 mg/OD); 4) not simultaneously participating in any interventional study. ^**^For 6 patients, if the baseline (BL) dose level is recorded as 'other,' use the closest dose of either 60 mg or 30 mg.

^#^Numbers are referring to the 1y-interim cut dated on 1 Feb 2024.

**Supplementary Figure S2.** Proportion of patients with hypertension or dyslipidemia at baseline

^*^Based on full analysis set (FAS), N=4877.

**Supplementary Figure S3.** Persistence patterns of edoxaban treatment A) switching pattern, B) suspension and discontinuation, C) continuing edoxaban use

**
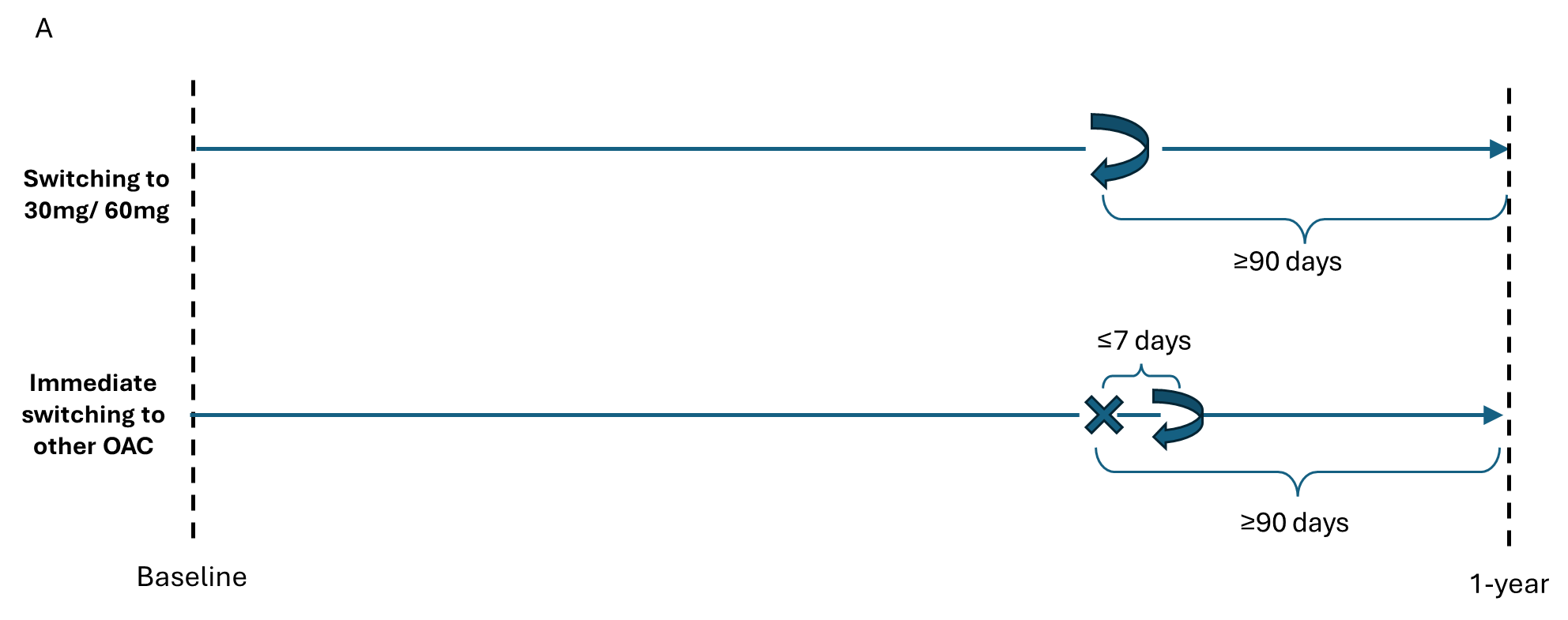
**

**
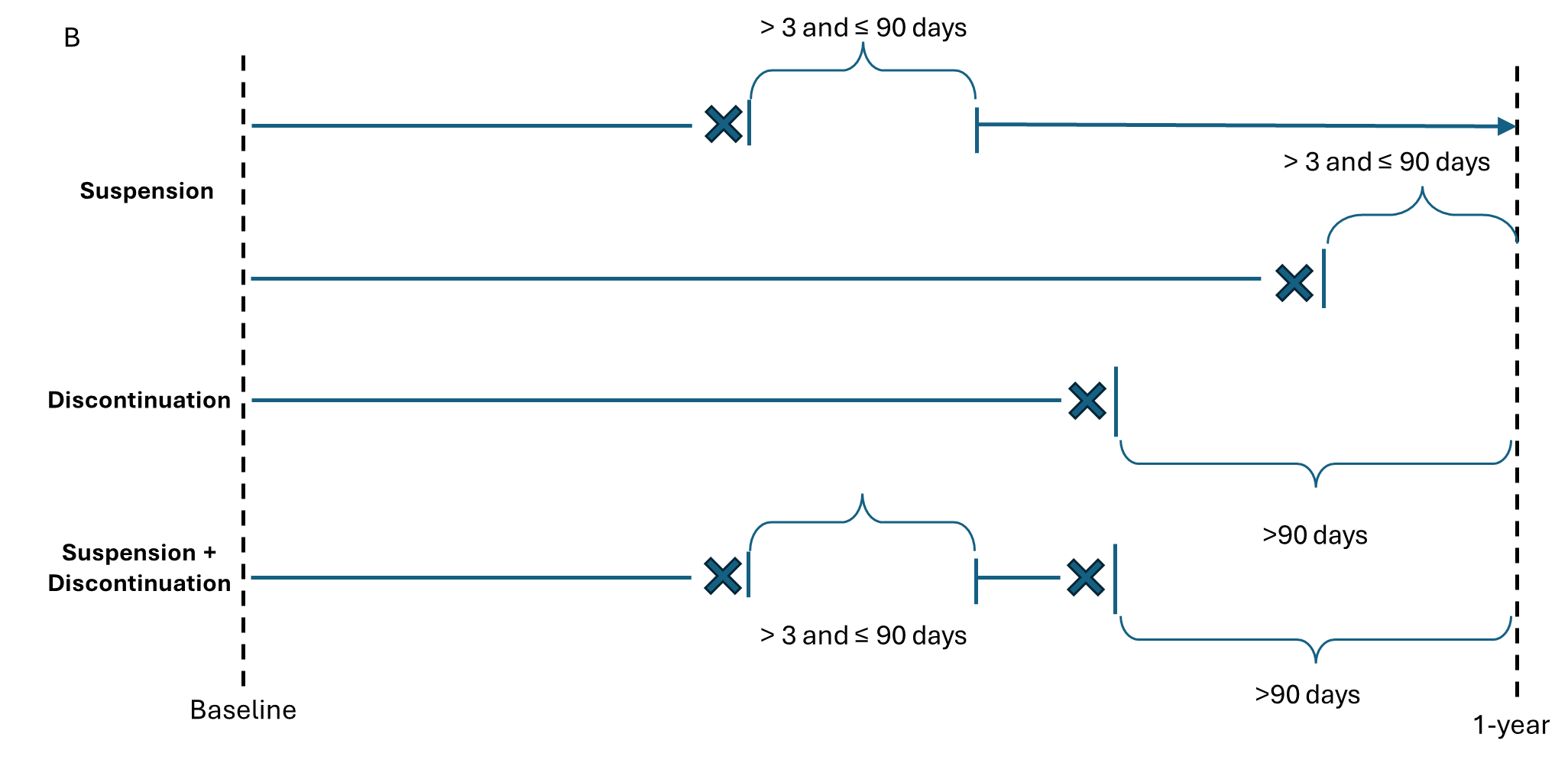
**

**
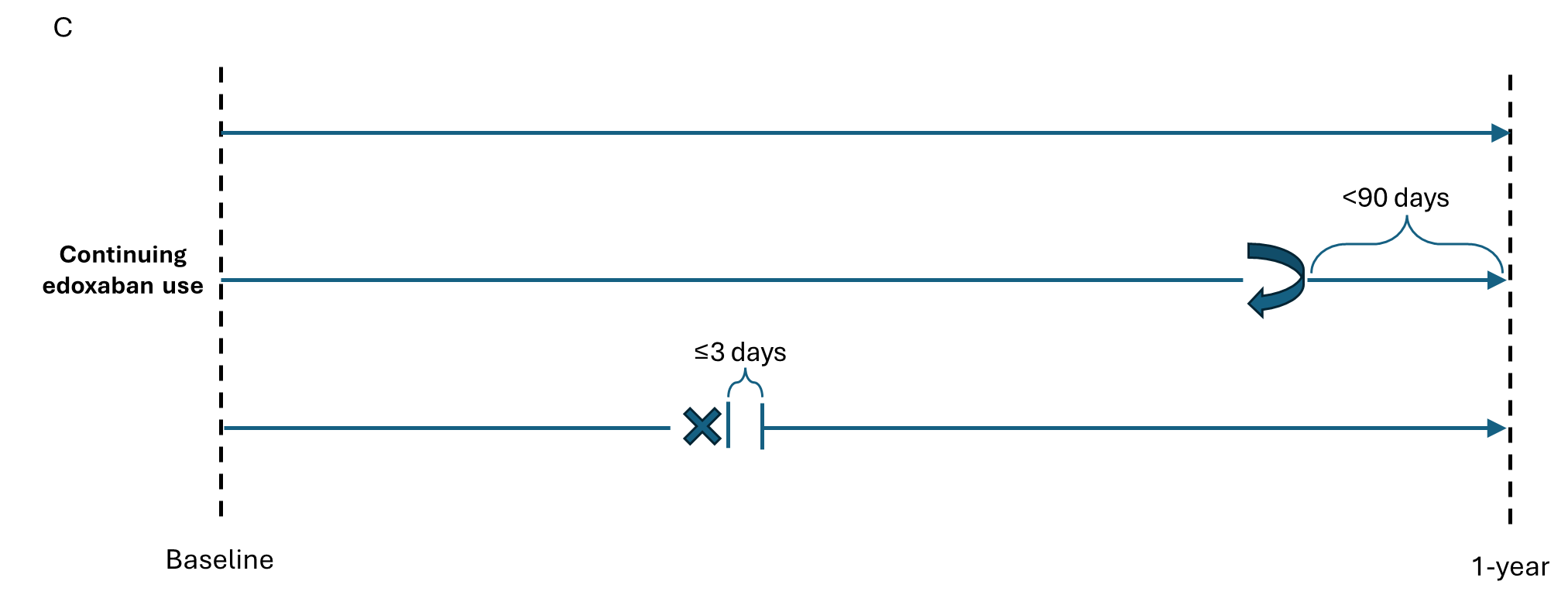
**

**Supplementary Figure S4.** Clinical outcomes in the 1-year follow-up of ETNA-AF-China patients dosed as (A) Non-recommended 30 mg vs recommended 60 mg, (B) Non-recommended 60 mg vs recommended 30 mg in accordance with the label^*^


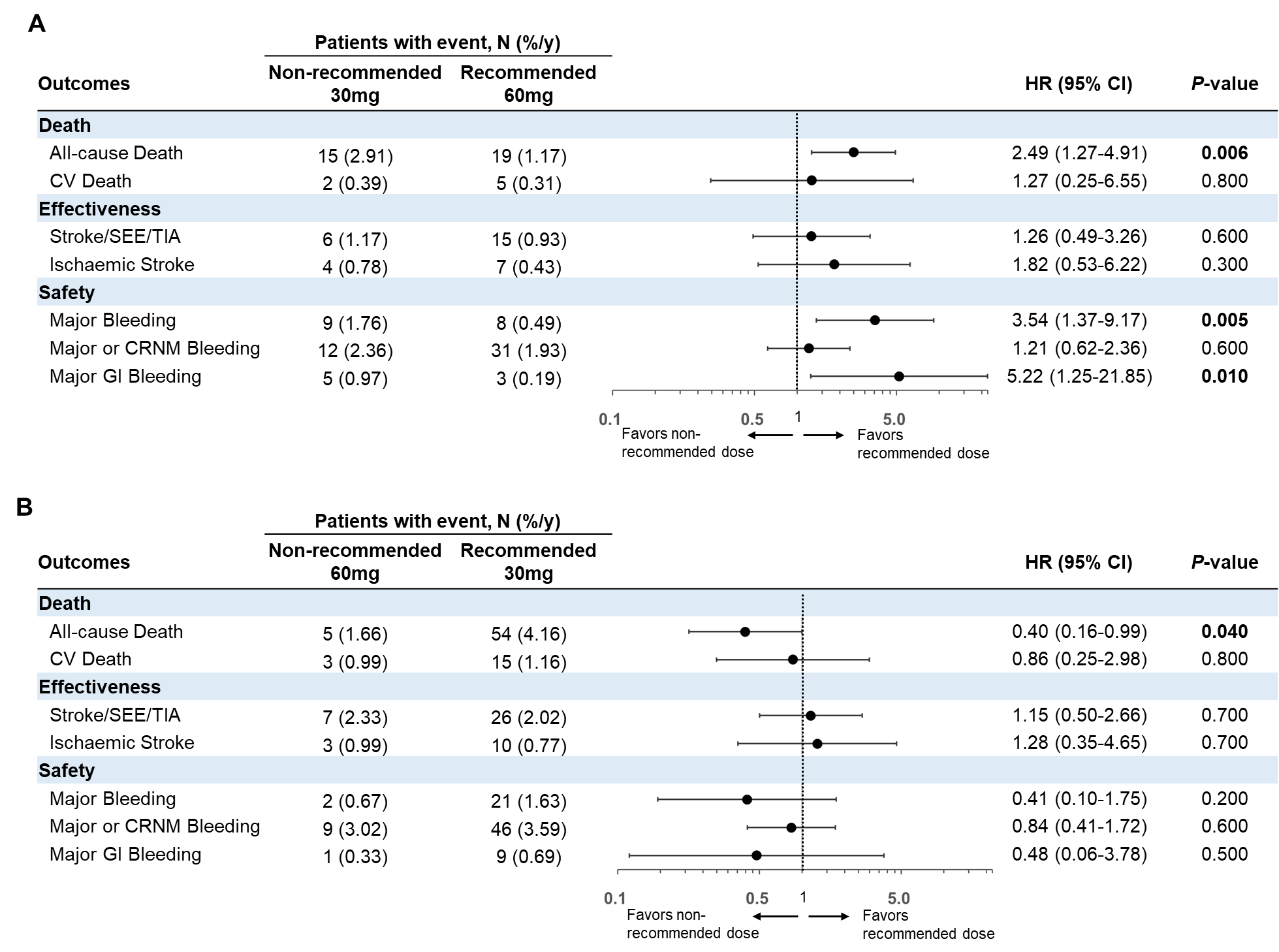


Forest plots showing HR (95% CI) of outcomes in using univariable Cox model.

^*^Calculated based on judgeable patients (*n* = 4192), not including 685 patients with non-judgable dosing.

HR, hazard ratio; CI, confidence interval; LVEF, left ventricular ejection fraction; SEE, systemic embolic event; TIA, transient ischaemic attack.

**Supplementary Figure S5.** Clinical outcomes of (A) acute coronary syndrome and (B) myocardial infarction in the 1-year follow-up of ETNA-AF-China patients by AF type

| 1-year clinical events, N (%/year) | **Paroxysmal** | **Persistent (>7d)** | **Long-standing persistent** | **Permanent** | **P value^#^** |
| --- | --- | --- | --- | --- | --- |
| ACS | 14 (0.89%) | 10 (0.7%) | 7 (0.98%) | 6 (1.02%) | 0.87 |
| MI | 5 (0.32%) | 1 (0.07%) | 1 (0.14%) | 4 (0.68%) | 0.08 |

^*^Based on full analysis set (FAS), N=4877.

^#^ Chi-square test was used for comparison on ACS events; Fisher's Exact Test was used for comparison on MI events.

ACS, acute coronary syndrome; AF, atrial fibrillation; MI, myocardial infarction

**Supplementary Table S1.** Comorbidities and related treatment of edoxaban-treatment patients with AF at baseline

|  | **Total** | **60 mg** | **30 mg** | ***P* value** |
| --- | --- | --- | --- | --- |
|  | **[n=4877]^*^ (100%)** | **[n=2650] (54.3%)** | **[n=2227] (45.7%)** |  |
| **COPD, n (%)** | 234/4877 (4.8) | 109/2650 (4.1) | 125/2227 (5.6) | 0.02 |
| **Treatment of COPD** | 118/234 (50.4) | 48/109 (44) | 70/125 (56) | 0.09 |
| **Hypertension, n (%)** | 3581/4877 (73.4) | 2010/2650 (75.8) | 1571/2227 (70.5) | <0.001 |
| **Treatment of hypertension** | 3194/3581 (89.2) | 1806/2010 (89.9) | 1388/1571 (88.4) | 0.16 |
| **Dyslipidaemia, n (%)** | 1225/4877 (25.1) | 723/2650 (27.3) | 502/2227 (22.5) | <0.001 |
| **Treatment of dyslipidaemia** | 996/1225 (81.3) | 585/723 (80.9) | 411/502 (81.9) | 0.73 |
| **Statins** | 924/1225 (75.4) | 545/723 (75.4) | 379/502 (75.5) | 0.99 |
| **Diabetes mellitus, n (%)** | 1284/4877 (26.3) | 737/2650 (27.8) | 547/2227 (24.6) | 0.01 |
| **Treatment** |  |  |  |  |
| **Insulin** | 148/1284 (11.5) | 88/737 (11.9) | 60/547 (11) | 0.89 |
| **Diet** | 56/1284 (4.4) | 30/737 (4.1) | 26/547 (4.8) | 0.52 |
| **Oral antidiabetic** | 844/1284 (65.7) | 496/737 (67.3) | 348/547 (63.6) | 0.91 |

^*^Based on full analysis set (FAS).

COPD, chronic obstructive pulmonary disease.

**Supplementary Table S2.** Univariable Cox regression analysis for predictors of all-cause death

| Variable | HR (95% CI) | *P* value |
| --- | --- | --- |
| Age | 1.07 (1.05-1.10) | <0.001 |
| By age subgroup |  |  |
| ≥65–<75 vs <65 years | 1.13 (0.59-2.14) | 0.71 |
| ≥75–<85 vs <65 years | 2.27 (1.24-4.18) | 0.008 |
| ≥85 vs <65 years | 6.94 (3.45-13.95) | <0.001 |
| BMI |  |  |
| <18.5 vs ≥18.5–<25 | 4.77 (2.54-8.95) | <0.001 |
| ≥25 vs ≥18.5–<25 | 0.70 (0.46-1.07) | 0.10 |
| By CrCl |  |  |
| ≥50–<80 vs ≥80 mL/min | 1.13 (0.63-2.03) | 0.68 |
| ≥30–<50 vs ≥80 mL/min | 2.44 (1.33-4.50) | 0.004 |
| <30 vs ≥80 mL/min | 10.52 (5.42-20.42) | <0.001 |
| CHA_2_DS_2_-VASc Score |  |  |
| ≥2–≤3 vs <2 | 0.92 (0.48-1.77) | 0.81 |
| ≥4 vs <2 | 2.16 (1.15-4.06) | 0.02 |
| HAS-BLED Score |  |  |
| ≥2–≤3 vs <2 | 1.59 (1.01-2.51) | 0.05 |
| ≥4 vs <2 | 0.87 (0.42-1.80) | 0.71 |
| Type of AF |  |  |
| Persistent vs paroxysmal | 2.34 (1.32-4.17) | 0.004 |
| Long-standing persistent vs paroxysmal | 2.86 (1.52-5.39) | 0.001 |
| Permanent vs paroxysmal | 3.96 (2.14-7.34) | <0.001 |
| Perceived frailty | 3.97 (2.40-6.57) | <0.001 |
| COPD | 3.06 (1.71-5.49) | <0.001 |
| Hypertension | 0.75 (0.49-1.14) | 0.18 |
| Heart failure (derived) | 2.37 (1.54-3.65) | <0.001 |
| Diabetes mellitus | 1.71 (1.14-2.56) | 0.009 |
| Not treated with insulin | 1.27 (0.77-2.08) | 0.35 |
| Treated with insulin | 2.36 (1.02-5.46) | 0.04 |
| Dyslipidaemia | 0.55 (0.32-0.95) | 0.03 |
| Valvular disease | 2.25 (1.20-4.21) | 0.01 |
| History of ischaemic stroke | 1.84 (1.03-3.30) | 0.04 |
| Economic region |  |  |
| Northeast vs East | 2.73 (1.48-5.00) | 0.001 |
| Middle vs East | 1.75 (1.11-2.78) | 0.02 |
| West vs East | 2.11 (1.09-4.07) | 0.03 |

^*^Based on full analysis set (FAS), N=4877. AF, atrial fibrillation; BMI, body mass index; COPD, chronic obstructive pulmonary disease; CI, confidence interval; CrCl, creatinine clearance; HR, hazard ratio.

**Supplementary Table S3.** Univariable Cox regression analysis for predictors of cardiovascular death

| Variable | HR (95% CI) | *P* value |
| --- | --- | --- |
| Age | 1.07 (1.02-1.12) | 0.008 |
| By age subgroup |  |  |
| ≥65–<75 vs <65 years | 0.56 (0.18-1.75) | 0.32 |
| ≥75–<85 vs <65 years | 1.46 (0.54-3.95) | 0.46 |
| ≥85 vs <65 years | 3.58 (1.01-12.68) | 0.05 |
| Body weight | 0.96 (0.93-1.00) | 0.03 |
| BMI |  |  |
| <18.5 vs ≥18.5–<25 | 2.71 (0.62-11.84) | 0.19 |
| ≥25 vs ≥18.5–<25 | 0.64 (0.29-1.43) | 0.28 |
| By CrCl |  |  |
| ≥50–<80 vs ≥80 mL/min | 0.68 (0.17-2.72) | 0.58 |
| ≥30–<50 vs ≥80 mL/min | 4.12 (1.27-13.37) | 0.02 |
| <30 vs ≥80 mL/min | 13.88 (3.73-51.72) | <0.001 |
| Type of AF |  |  |
| Persistent vs paroxysmal | 3.05 (0.97-9.57) | 0.06 |
| Long-standing persistent vs paroxysmal | 2.76 (0.74-10.29) | 0.13 |
| Permanent vs paroxysmal | 4.71 (1.38-16.08) | 0.01 |
| Perceived frailty | 4.76 (1.99-11.39) | <0.001 |
| Hypertension | 0.38 (0.18-0.81) | 0.01 |
| Heart failure (derived) | 3.42 (1.57-7.47) | 0.002 |
| Diabetes mellitus | 1.39 (0.63-3.10) | 0.42 |
| Not treated with insulin | 1.25 (0.49-3.14) | 0.64 |
| Treated with insulin | 4.08 (1.20-13.87) | 0.02 |
| Dyslipidaemia | 0.35 (0.11-1.17) | 0.09 |
| Valvular disease | 3.19 (1.10-9.27) | 0.03 |

^*^Based on full analysis set (FAS), N=4877. AF, atrial fibrillation; BMI, body mass index; CI, confidence interval; CrCl, creatinine clearance; HR, hazard ratio.

**Supplementary Table S4.** Univariable Cox regression analysis for predictors of stroke/systemic embolic events/ transient ischaemic attack

| Variable | HR (95% CI) | *P* value |
| --- | --- | --- |
| Age | 1.04 (1.01-1.07) | 0.01 |
| By age subgroup | | |
| ≥65–<75 vs <65 years | 3.97 (1.55-10.13) | 0.004 |
| ≥75–<85 vs <65 years | 3.20 (1.20-8.52) | 0.02 |
| ≥85 vs <65 years | 3.25 (0.78-13.62) | 0.11 |
| By CrCl | | |
| ≥50–<80 vs ≥80 mL/min | 2.30 (1.05-5.07) | 0.04 |
| ≥30–<50 vs ≥80 mL/min | 2.98 (1.24-7.19) | 0.02 |
| <30 vs ≥80 mL/min | 7.05 (2.31-21.56) | 0.001 |
| CHA_2_DS_2_-VASc Score | | |
| ≥2–≤3 vs <2 | 2.85 (0.87-9.41) | 0.09 |
| ≥4 vs <2 | 5.16 (1.57-16.95) | 0.007 |
| HAS-BLED Score | | |
| ≥2–≤3 vs <2 | 3.41 (1.60-7.27) | 0.001 |
| ≥4 vs <2 | 3.70 (1.53-8.93) | 0.004 |
| Perceived frailty | 2.25 (1.06-4.79) | 0.03 |
| Heart failure (derived) | 2.16 (1.24-3.76) | 0.007 |
| History of ischaemic stroke | 2.08 (1.03-4.21) | 0.04 |
| History of TIA | 2.98 (1.08-8.22) | 0.03 |

^*^Based on full analysis set (FAS), N=4877. Stroke/SEE/TIA refers to the first occurrence of event of all stroke or SEE or TIA.

CI, confidence interval; CrCl, creatinine clearance; HR, hazard ratio; TIA, transient ischaemic attack.

**Supplementary Table S5.** Univariable Cox regression analysis for predictors of major bleeding.

| Variable | HR (95% CI) | *P* value |
| --- | --- | --- |
| Age | 1.07 (1.03-1.11) | <0.001 |
| By age subgroup | | |
| ≥65–<75 vs <65 years | 3.39 (1.00-11.49) | 0.05 |
| ≥75–<85 vs <65 years | 5.31 (1.58-17.88) | 0.007 |
| ≥85 vs <65 years | 7.07 (1.58-31.61) | 0.010 |
| BMI | 0.95 (0.88-1.04) | 0.27 |
| <18.5 vs ≥18.5–<25 | 5.00 (1.88-13.32) | 0.001 |
| ≥25 vs ≥18.5–<25 | 0.97 (0.52-1.81) | 0.93 |
| By CrCl | | |
| ≥50–<80 vs ≥80 mL/min | 4.09 (1.20-13.87) | 0.02 |
| ≥30–<50 vs ≥80 mL/min | 7.95 (2.26-27.88) | 0.001 |
| <30 vs ≥80 mL/min | 18.40 (4.40-77.03) | <0.001 |
| CHA_2_DS_2_-VASc Score |  |  |
| ≥2–≤3 vs <2 | 6.32 (0.85-47.12) | 0.07 |
| ≥4 vs <2 | 12.22 (1.65-90.48) | 0.01 |
| HAS-BLED Score | | |
| ≥2–≤3 vs <2 | 3.23 (1.34-7.75) | 0.009 |
| ≥4 vs <2 | 3.39 (1.21-9.53) | 0.02 |
| Type of AF | | |
| Persistent vs paroxysmal | 4.28 (1.74-10.50) | 0.002 |
| Long-standing persistent vs paroxysmal | 2.57 (0.86-7.66) | 0.09 |
| Permanent vs paroxysmal | 4.05 (1.44-11.37) | 0.008 |
| Perceived frailty | 2.90 (1.34-6.27) | 0.007 |
| Heart failure (derived) | 2.12 (1.10-4.11) | 0.03 |

^*^Based on full analysis set (FAS), N=4877. AF, atrial fibrillation; BMI, body mass index; CI, confidence interval; CrCl, creatinine clearance; HR, hazard ratio.

**Step-wise multivariable cox regression analysis of recommended/non-recommended doses**

In the model of non-recommended 60 mg versus recommended 30 mg dose of edoxaban, permanent AF type (HR: 3.35, 95% CI: 1.53–7.34; *P* = 0.002), BMI < 18.5 (HR: 2.65, 95% CI: 1.35–5.22; *P* = 0.005), male gender (HR: 2.85, 95% CI: 1.64–4.94; *P* < 0.001), and perceived frailty (HR: 3.72, 95% CI: 1.96–7.09; *P* <0.001) were significant predictors of all-cause death. Diabetes mellitus (HR: 2.18, 95% CI: 1.21–3.93; *P* = 0.010), valvular disease (HR: 2.26, 95% CI: 1.04-4.91; *P* = 0.041), history of major bleeding (HR: 5.38, 95% CI: 1.59–18.24; *P* = 0.007), and economic regions of Northeast (HR: 3.52, 95% CI: 1.38–8.99; *P* = 0.008) were also associated with all-cause death. The predictors of CV death in patients prescribed with the non-recommended 60 mg dose of edoxaban included perceived frailty (HR: 5.49, 95% CI: 1.94–15.53; *P* = 0.001), valvular disease (HR: 3.78, 95% CI: 1.21–11.77; *P* = 0.022), history of major bleeding (HR: 15.13, 95% CI: 1.73–132.32; *P* = 0.014). A BMI <18.5 (HR: 4.65, 95% CI: 1.55–12.98; *P* = 0.006) and hypertension (HR: 4.39, 95% CI: 1.01–19.08; *P* = 0.049) were predictors of major bleeding in patients who were prescribed the non-recommended 60 mg dose of edoxaban. The predictor of stroke/TIA/SEE in the non-recommended 60 mg dose group was HF (HR: 2.23, 95% CI: 1.06–4.69; *P* = 0.035).

In patients who were prescribed the non-recommended 30-mg dose of edoxaban, long-standing persistent AF type (HR: 5.28, 95% CI: 1.65–16.90 *P* = 0.005), permanent AF (HR: 4.21, 95% CI: 1.22–14.47; P = 0.023), and HF (HR: 2.74, 95% CI: 1.36–5.52; *P* = 0.005) were identified as predictors of all-cause death. In addition, diabetes mellitus (HR: 2.36, 95% CI: 1.19–4.66; *P* = 0.014) and dyslipidaemia (HR: 0.28, 95% CI: 0.09–0.92; *P* = 0.037) were also associated with all-cause death. In the non-recommended 30 mg group, HF (HR: 7.67, 95% CI: 1.67–35.25; *P* = 0.009) was identified as a predictor of CV death. In the non-recommended 30-mg group, HAS-BLED score (HR: 1.87, 95% CI: 1.17–2.99; *P* = 0.009) was associated with major bleeding, in addition to persistent AF type (HR: 5.99, 95% CI: 1.32-27.10; *P* = 0.020), permanent AF type (HR: 5.75, 95% CI: 1.04–31.77; *P* = 0.045), and diabetes mellitus (HR: 3.11, 95% CI: 1.20–8.09; *P* = 0.020). HAS-BLED score (HR: 2.30, 95% CI: 1.54–3.44; *P* < 0.001) was also identified as a significant predictor of stroke/TIA/SEE in those with non-recommended 30 mg (**Supplementary Table S6)**.

**Supplementary Table S6.** Multivariable cox regression analysis^#^ of recommended/ non-recommended doses and characteristics associated with (A) all-cause death, (B) cardiovascular death, (C) major bleeding and (D) stroke/TIA/SEE outcomes during the 1-year follow-up^*^.

|  | **HR (95% CI)** | ***P*-value** |  | **HR (95% CI)** | ***P*-value** |
| --- | --- | --- | --- | --- | --- |
| **All cause death** | | | | | |
| **Non-recommended 60 mg vs recommended 30 mg** | 0.52 (0.21-1.39) | 0.203 | **Non-recommended 30 mg vs recommended 60 mg** | 2.43 (1.22-4.82) | **0.011** |
| **Type of AF** |  |  | **Type of AF** |  |  |
| Persistent vs Paroxysmal | 1.78 (0.84-3.79) | 0.134 | Persistent vs Paroxysmal | 2.91 (0.94-9.01) | 0.064 |
| Long-standing persistent vs Paroxysmal | 2.26 (0.95-5.37) | 0.064 | Long-standing persistent vs Paroxysmal | 5.28 (1.65-16.90) | **0.005** |
| Permanent vs Paroxysmal | 3.35 (1.53-7.34) | **0.002** | Permanent vs Paroxysmal | 4.21 (1.22-14.47) | **0.023** |
| **BMI** |  |  | - | | |
| <18.5 vs ≥18.5–<25 | 2.65 (1.35-5.22) | **0.005** | - | | |
| ≥ 25 vs ≥18.5–<25 | 0.94 (0.44-2.02) | 0.872 | - | | |
| **Male gender vs female** | 2.85 (1.64-4.94) | **<0.001** | - | | |
| **Perceived frailty** | 3.72 (1.96-7.09) | **<0.001** | - | | |
| **Diabetes mellitus** | 2.18 (1.21-3.93) | **0.010** | **Diabetes mellitus** | 2.36 (1.19-4.66) | **0.014** |
| **-** | | | **Dyslipidaemia** | 0.28 (0.09-0.92) | **0.037** |
| **Valvular disease** | 2.26 (1.04-4.91) | **0.041** | **-** | | |
| **-** | | | **Heart failure (derived)** | 2.74 (1.36-5.52) | **0.005** |
| **-** | | | **History of ischaemic stroke** | 2.13 (0.82-5.51) | 0.120 |
| **History of major bleeding** | 5.38 (1.59-18.24) | **0.007** | **-** | | |
| **Economic region** |  |  | **-** | | |
| Northeast vs East | 3.52 (1.38-8.99) | **0.008** | **-** | | |
| Middle vs East | 1.48 (0.82-2.69) | 0.193 | **-** | | |
| West vs East | 0.71 (0.27-1.87) | 0.490 | **-** | | |
| **CV death** | | | | | |
| **Non-recommended 60 mg vs recommended 30 mg** | 1.36 (0.38-4.87) | 0.640 | **Non-recommended 30 mg vs recommended 60 mg** | 1.25 (0.24-6.45) | 0.791 |
| **Perceived frailty** | 5.49 (1.94-15.53) | **0.001** | **-** | | |
| **Valvular disease** | 3.78 (1.21-11.77) | **0.022** | **-** | | |
| **History of major bleeding** | 15.13 (1.73-132.32) | **0.014** | **-** | | |
| **CHA2DS2-VASc Score** | 0.75 (0.52-1.09) | 0.130 | **-** | | |
| **-** | | | **Heart failure (derived)** | 7.67 (1.67-35.25) | **0.009** |
| **Major bleeding** | | | | | |
| **Non-recommended 60 mg vs recommended 30 mg** | 0.54 (0.12-2.38) | 0.418 | **Non-recommended 30 mg vs recommended 60 mg** | 2.90 (1.10-7.66) | **0.032** |
| **BMI** |  |  | - |  |  |
| <18.5 vs ≥18.5–<25 | 4.65 (1.55-12.98) | **0.006** | - |  |  |
| ≥ 25 vs ≥18.5–<25 | 1.83 (0.69-4.82) | 0.224 | - |  |  |
| **Hypertension** | 4.39 (1.01-19.08) | **0.049** | - |  |  |
| **Perceived frailty** | 2.31 (0.89-6.00) | 0.085 | **Perceived frailty** | 1.05 (0.23-4.81) | 0.952 |
| - | | | **Type of AF** |  |  |
| - | | | Persistent vs Paroxysmal | 5.99 (1.32-27.10) | **0.020** |
| - | | | Permanent vs Paroxysmal | 5.75 (1.04-31.77) | **0.045** |
| - | | | **HAS-BLED Score** | 1.87 (1.17-2.99) | **0.009** |
| - | | | **Diabetes mellitus** | 3.11 (1.20-8.09) | **0.020** |
| **Stroke/TIA/SEE** | | | | | |
| **Non-recommended 60 mg vs recommended 30 mg** | 1.24 (0.54-2.87) | 0.610 | **Non-recommended 30 mg vs recommended 60 mg** | 1.01 (0.39-2.61) | 0.983 |
| **Heart failure (derived)** | 2.23 (1.06-4.69) | **0.035** | - | | |
| **HAS-BLED Score** | 1.37 (0.92-2.03) | 0.118 | **HAS-BLED Score** | 2.30 (1.54-3.44) | **<0.001** |

^#^Multivariable cox regression analysis by fixing non-recommended 60 mg vs recommended 30 mg (left column), non-recommended 30 mg vs recommended 60 mg (right column) and all characteristics in a stepwise regression model. *Based on judgeable patients (N = 4192), not including 685 patients with non-judgable dosing.

**Supplementary Table S7. Outcomes reported in ETNA-AF-China and other prospective registries**

| Event | ETNA-AF-China  (n = 4877) | XANAP (Kim et al., 2018)  (n=2733) | XAPASS (Ikeda et al., 2019)  (n = 9578) | GLORIA-AF (Mazurek et al., 2019) (n = 654) | SAKURA AF (Okumura et al., 2018)  (n = 3157) | GARFIELD-AF (Bassand et al., 2019) (n = 52,014) |
| --- | --- | --- | --- | --- | --- | --- |
| Study Type | Single-arm, prospective, observational, post-authorisation | Single-arm, prospective, observational, post-authorisation | Single-arm, prospective, observational, post-authorisation | Phase 2, global, prospective, registry | Prospective, observational, registry | Global, prospective, observational |
| NOAC | Edoxaban (100%^&^) | Rivaroxaban (100%) | Rivaroxaban (100%) | Dabigatran (100%) | NOAC (51.8%)  Wafarin (48.2%) | NOAC ± AP (27.5%)  VKA ± AP (39.3%)  AP only (21.0%)  None (12.2%) |
| Region | Chinese Mainland | Asian Pacific: Hong Kong, Indonesia, Malaysia, Pakistan, Philippines, Singapore, South  Korea, Taiwan, Thailand, and Vietnam | Japan | Asia: China, Hong Kong, Japan, Russia, Singapore, South Korea, Taiwan | Japan | 35 countries in Asia, Europe, Latin America, North America, and other GARFIELD-AF countries |
| Follow-up duration, years | 1 year | 1 year | 1 year | 2 years | 1 year | Over 12 months |
| Event rates | %/year (95% CI)*† | Events/100PY (95% CI)* | Events/100PY (95% CI) | Crude incidence rate/100PY  (95% CI) | %/year (95% CI)  NOAC | Events/100PY  (95% CI) |
| Stroke | 1.11 (0.84-1.47) | 1.9 (1.3–2.6)‡ | 1.6 (1.4–1.9)‡ | 0.90 (0.39–1.77) | 1.2 (0.7–1.8)§ | 1.3 (1.2–1.4)¶ |
| Major bleeding | 1.04 (0.78-1.39) | 1.5 (1.0–2.1) | 1.8 (1.5–2.1) | 0.90 (0.39–1.77) | 0.5 (0.3–1.1) | 0.8 (0.8–0.9)# |
| All-cause death | 2.3 (1.89-2.8) | 2.0 (1.4–2.7) | 2.5 (2.1–2.8) | 1.12 (0.54–2.06) | 2.1 (1.5–2.9) | 4.3 (4.2–4.5) |
| CV death | 0.62 (0.43-0.91) | – | – | 0.11 (0.00–0.62)‖ | – | 1.6 (1.5–1.7) |

^&^Prescription at baseline;

*Adjudicated events;

†Outcomes are reported for the full analysis set except for bleeding outcomes, which are reported for on-treatment patients;

§Composite effectiveness endpoint (stroke/systemic embolism);

‡Composite effectiveness endpoint (Stroke/non-CNS SE);

‖Vascular death;

¶Non-haemorrhagic stroke/SE (defined as the combined endpoints of ischaemic stroke, unknown-type stroke, SE, and TIA);

#Major bleed/haemorrhagic stroke;

AP, antiplatelet; CI, confidence interval; CNS, central nervous system; CV, cardiovascular; NOAC, non-vitamin K antagonist oral anticoagulant; VKA, vitamin K antagonists; PY, patient-years.

**References:**

Bassand, J.-P., Virdone, S., Goldhaber, S. Z., Camm, A. J., Fitzmaurice, D. A., Fox, K. A. A., et al. (2019). Early Risks of Death, Stroke/Systemic Embolism, and Major Bleeding in Patients With Newly Diagnosed Atrial Fibrillation: Results From the GARFIELD-AF Registry. *Circulation* 139, 787–798. doi: 10.1161/CIRCULATIONAHA.118.035012

Ikeda, T., Ogawa, S., Kitazono, T., Nakagawara, J., Minematsu, K., Miyamoto, S., et al. (2019). Real-world outcomes of the Xarelto Post-Authorization Safety & Effectiveness Study in Japanese Patients with Atrial Fibrillation (XAPASS). *Journal of Cardiology* 74, 60–66. doi: 10.1016/j.jjcc.2019.01.001

Kim, Y., Shim, J., Tsai, C., Wang, C., Vilela, G., Muengtaweepongsa, S., et al. (2018). XANAP: A real‐world, prospective, observational study of patients treated with rivaroxaban for stroke prevention in atrial fibrillation in Asia. *Journal of Arrhythmia* 34, 418–427. doi: 10.1002/joa3.12073

Mazurek, M., Teutsch, C., Diener, H.-C., Dubner, S. J., Halperin, J. L., Ma, C.-S., et al. (2019). Safety and effectiveness of dabigatran at 2 years: Final outcomes from Phase II of the GLORIA-AF registry program. *American Heart Journal* 218, 123–127. doi: 10.1016/j.ahj.2019.08.012

Okumura, Y., Yokoyama, K., Matsumoto, N., Tachibana, E., Kuronuma, K., Oiwa, K., et al. (2018). Three-Year Clinical Outcomes Associated With Warfarin vs. Direct Oral Anticoagulant Use Among Japanese Patients With Atrial Fibrillation ― Findings From the SAKURA AF Registry ―. *Circ J* 82, 2500–2509. doi: 10.1253/circj.CJ-18-0535
